# Dual-Functional Organosilicon Additives Containing Methacrylate and Trimethoxysilyl Groups Enhancing Impact Toughness of Polylactide (PLA): Structure–Property Relationship

**DOI:** 10.3390/ma18122903

**Published:** 2025-06-19

**Authors:** Julia Głowacka, Miłosz Frydrych, Eliza Romańczuk-Ruszuk, Yi Gao, Hui Zhou, Robert E. Przekop, Bogna Sztorch

**Affiliations:** 1 Center for Advanced Technologies, Adam Mickiewicz University Poznan, Uniwersytetu Poznanskiego 10, 61-614 Poznan, Poland; julia.glowacka@amu.edu.pl (J.G.); frydrych@amu.edu.pl (M.F.); rprzekop@amu.edu.pl (R.E.P.); 2 Faculty of Chemistry, Adam Mickiewicz University Poznan, Uniwersytetu Poznanskiego 8, 61-614 Poznan, Poland; 3 Institute of Biomedical Engineering, Faculty of Mechanical Engineering, Bialystok University of Technology, Wiejska 45C, 15-351 Bialystok, Poland; e.romanczuk@pb.edu.pl; 4 Key Laboratory for Thermal Science and Power Engineering of Ministry of Education, Beijing Key Laboratory of CO_2_ Utilization and Reduction Technology, Department of Energy and Power Engineering, Tsinghua University, Beijing 100084, China; 18gy@mail.tsinghua.edu.cn (Y.G.); huizhou@tsinghua.edu.cn (H.Z.)

**Keywords:** polylactide (PLA), octaspherosilicate, cyclosiloxane, impact resistance, rheology, fracture behavior, nanocomposites

## Abstract

The demands of the green economy necessitate modern polymer materials that are not only environmentally friendly but also durable and capable of long service life. Bio-based polylactide (PLA) polyesters have gained significant traction in various industrial markets; however, their application in specialized sectors is hindered by high brittleness. This study extensively examines the effects of 1–5% of synthetically obtained tetracyclosiloxane (CS) and octaspherosilicate (OSS) derivatives with methacrylate (MA) and trimethoxysilyl (TMOS) groups as functional modifiers for PLA. The research provides a detailed characterization of PLA/CS and PLA/OSS materials, including a comparative analysis of mechanical properties such as tensile, flexural, and dynamic resistance. Notably, incorporating 5% CS-2MA-2TMOS into PLA resulted in a remarkable 104% increase in impact resistance. The study further evaluates the influence of these modifications on thermal properties (DSC, TGA), heat deflection temperature (HDT), and surface character (WCA). The miscibility between the organosilicon additives and PLA was assessed using oscillatory rheometry and SEM-EDS analysis. The melt-rheology analysis explained the mechanisms behind the interaction between the CS and OSS additives with the PLA matrix, highlighting their lubricating effects on the melt flow behavior. The study was complemented by XRD structural analysis and verification of the structure of PLA-based materials by optical microscopy and SEM analysis, demonstrating a plasticizing effect and uniform distribution of the modifiers. The findings strongly suggest that, even at low concentrations, organosilicon additives serve as effective impact modifiers for PLA.

## 1. Introduction

The shift toward sustainable development drives demand for biobased, eco-friendly polymers, aiming to reduce environmental impact by replacing petrochemical polymers with alternative materials. Polyactide (PLA), a thermoplastic polyester derived from natural sources like corn, sugarcane, and starch-based feedstocks, is produced through biotechnological processes involving lactic acid fermentation and ring-opening polymerization, offering an eco-friendly alternative to traditional refinery methods and gaining popularity with rising environmental awareness [1,2,3,4]. Now, PLA is used in many branches of industry because of its good melt flowability (processability) and biocompatibility, from the packing industry [5,6] to more advanced biomedical applications (stents, tissue regenerative medicine, implants) [7,8,9] and then the production of more complicated elements in additive manufacturing [10,11] or target material for undemanding parts in the automotive industry. Despite its biodegradability, the use of PLA remains limited due to concerns over its high brittleness, low ductility, and insufficient toughness. Driven by scientific curiosity and practical needs, researchers aim to enhance PLA’s properties, focusing on reducing brittleness and improving resistance to high temperatures and mechanical stress to make it more suitable for advanced technical applications [12,13,14].

The use of organosilicon compounds to modify thermoplastics has been well-documented, with their nanoparticles serving multiple roles in polymer nanocomposites, including enhancing mechanical [15,16,17,18], rheological [19,20,21,22], and thermal properties [23,24], as well as fire retardants [25,26] and UV protectors [24,27], and compatibilizers of surface interaction between polymer matrix and fillers [28]. Silsesquioxanes and their subgroup octaspherosilicates (OSS) are among the most extensively studied inorganic–organic hybrid modifiers. The general formula for silsesquioxanes is [RSiO_3/2_]_n_, where R represents hydrogen, alkyl, alkene, aryl, or other organic substituents [29]. These compounds feature a unique cage-like structure, often described as a cube or polyhedron, due to the arrangement of silicon and oxygen atoms. Their distinct -Si-O-Si- core, combined with various functional groups on the peripheral atoms, makes them versatile additives for polymer modifications, allowing tailored properties based on the choice of organic substituents [30,31,32,33,34,35] and enabling diverse applications in materials science, nanotechnology, and chemistry [36,37,38]. Among the various derivatives of silsesquioxanes, methacrylate-functionalized polyhedral oligomeric silsesquioxane (MA-POSS) has emerged as a particularly versatile building block for the development of advanced polymer nanocomposites [39]. MA-POSS has garnered significant attention in advanced polymer materials for its reactive nature, which enhances mechanical [40], thermal [41], and optical properties [42], and it has successfully been incorporated into a wide range of polymers, including thermoplastics, thermosets, and elastomers [43,44,45]. The unique properties of MA-POSS, such as its low dielectric constant and ability to enhance the fire retardancy of polymers, make it a valuable additive for applications in the electronics, aerospace, and automotive industries [46,47]. Furthermore, the biocompatibility and low toxicity of MA-POSS have led to its exploration in biomedical applications, such as drug delivery systems and tissue engineering [48].

Cyclosiloxanes are a class of chemical compounds characterized by cyclic Si-O-Si rings, with a general formula of [RSiO]_n_, where R denotes the organic substituents and n signifies the number of repeating units in the cyclic structure [49]. Unlike caged structures, cyclosiloxanes allow for greater movement of Si-O-Si units within the ring, facilitating better compatibility with modifier and polymer molecules. Their unique arrangement combines flexibility, thermal stability, and chemical resistance, making them ideal candidates for enhancing the performance of various polymeric materials [50,51,52]. The unique properties of tetracyclosiloxane (CS), a specific type of cyclosiloxane, such as their low viscosity [53] and excellent compatibility with organic matrices [54], make them easy to incorporate into polymer systems, resulting in improved mechanical properties and enhanced durability [55]. Furthermore, during curing processes, tetracyclosiloxanes can form cross-linked networks, leading to the development of advanced materials with tailored characteristics suitable for specific applications [56]. In recent years, there has been a growing focus on the role of tetracyclosiloxanes in formulating silicone-based polymers [57], coatings [58], and liquid crystals [59]. These materials are used not only in industrial applications but also in consumer products, including electronics and healthcare items. The integration of tetracyclosiloxanes into polymer matrices can enhance several properties such as hydrophobicity [60], thermal resistance [61], and flame retardance [62], thereby expanding the functional capabilities of these materials. Some research has also focused on using tetracyclosiloxanes as supramolecular crystallization agents, but there has been no further investigation into the effect of these agents on PLA’s mechanical properties [63].

This work aims to develop durable and long-lasting PLA-based materials that can meet the demands of the green economy. By the synthetic functionalization of CS and OSS via hydrosilylation reactions, functional organosilicon additives were obtained and introduced into PLA by standard plastic processing methods. This paper focuses on the extensive study of tetracyclosiloxane and octaspherosilicate derivatives with methacrylate (MA) and trimethoxysilyl (TMOS) groups as functional modifiers of PLA. The research provides a detailed characterization of PLA/CS and PLA/OSS materials (effect of 1–5% organosilicon additives) on mechanical properties (impact resistance, flexural, and tensile properties), and further evaluates the influence of modifiers on thermal stability (TGA), changes in phase transformation temperatures and degree of crystallinity (DSC), heat deflection temperature (HDT), and surface properties (WCA). The miscibility between the filler and the polymer was assessed using oscillatory rheometry and SEM-EDS analysis. The melt-rheology analysis allowed us to explain the mechanisms behind the interaction between the CS and OSS additives with PLA, highlighting their lubricating effects on PLA melt flow behavior. The study is further supported by X-ray diffraction (XRD) analysis, and the morphology of the PLA-based materials was confirmed through optical microscopy and scanning electron microscopy (SEM) analysis, demonstrating a plasticizing effect and uniform distribution of the modifiers. Overall, the findings strongly suggest that organosilicon additives, even at low concentrations, effectively impact PLA properties.

## 2. Materials and Methods

### 2.1. Materials

Polylactide (PLA) Ingeo 2003D type was purchased from NatureWorks (Minnetonka, Minneapolis, MN, USA). Chemical compounds were purchased from the following sources: tetraethoxysilane (TEOS), chlorodimethylsilane, tetramethylammonium hydroxide (TMAH) 25 wt% methanol solution from ABCR, vinyltrimethoxysilane from Linegal Chemicals (Warsaw, Poland), chloroform-d, 2 wt% Karstedt’s catalyst xylene solution from Merck (Poznan, Poland), allyl methacrylate, P_2_O_5,_ and toluene from Avantor Performance Materials Poland S.A. (Gliwice, Poland), and 1,3,5,7-tetramethylcyclotetrasiloxane from Gelest Inc. (Morrisville, PA, USA). Toluene was degassed and dried by distilling it from P_2_O_5_ under an argon atmosphere.

### 2.2. Methods

Nuclear Magnetic Resonance (NMR) spectra for ^1^H, ^13^C, and ^29^Si were recorded at 25 °C on Bruker Ascend 400 and Ultra Shield 300 spectrometers (Bruker, Billerica, MA, USA) using CDCl_3_ as a solvent. The chemical shifts are reported in the ppm scale, and the residual solvent (CHCl_3_) signals were used as a reference for ^1^H and ^13^C.

A Charpy impact test (with no notch) was performed on an Instron Ceast 9050 impact machine (Instron, Norwood, MA, USA) according to PN-EN ISO 179 [64]. Standard ISO specimens 80 × 10 × 4 mm (type 1B) were chosen. For all the series, 7 measurements were performed. The average and standard deviation were determined for each measurement series.

Flexural tests were performed on a universal testing machine, INSTRON 5969 (Instron, Norwood, MA, USA), with a maximum load force of 50 kN. Specimens were prepared following the requirements of PN-EN ISO 178 [65]. For the test standard specimens, type 1B, 80 × 10 × 4 mm, were used. The traverse speed for measurements was set at 2 mm/min, and maximum deflection was equal to 6 mm. The average from 7 specimens and the standard deviation were determined for each measurement series.

For tensile strength tests, standard 1A injection-molded specimens were used according to the requirements of PN-EN ISO 527 [66]. The specimens obtained were tested on a universal testing machine, INSTRON 5969 (Instron, Norwood, MA, USA), with a maximum load force of 50 kN. The traverse speed for measurements was set at 5 mm/min. Seven measurements were performed. The average and standard deviation were determined for each measurement series.

Scanning electron microscopy (SEM) microphotographs were taken using a Quanta FEG 250 (FEI) high-resolution scanning electron microscope (Osaka, Japan) to analyze the microstructure and quality of the produced samples (at 5 kV). SEM with energy dispersive spectroscopy (SEM-EDS) analyses were recorded on a Phenom XL scanning electron microscope (at 15 kV, low vacuum).

Surface topography was analyzed under Digital Light Microscope Keyence VHX 7000 with 100× to 1000× VH-Z100T lens (Keyence International, Mechelen, Belgium). All the pictures were recorded with a VHX 7020 camera.

X-Ray diffraction (XRD) was performed using a powder diffractometer (SmartLab Rigaku, Tokyo, Japan) with a CuK alpha lamp, in the range of 3–100° (2θ), scan step 0.01, scan speed 4°/min. The crystallinity level (*X_c_*) was calculated using the following Equation (1):

(1) $$X_{c}=\frac{A_{c}}{A_{c}+A_{a}}$$

where

*X_c_* [%]—degree of crystallinity;

*A_c_*—crystallized area on the diffractogram;

*A_a_*—amorphous area on the diffractogram.

Thermogravimetry (TGA) was performed using a NETZSCH 209 F1 Libra gravimetric analyzer (Netzsch Group, Selb, Germany). Samples of 5 ± 0.2 mg were cut from each granulate and placed in Al_2_O_3_ crucibles. Measurements were conducted under nitrogen (flow of 20 mL/min) in the range of 30–950 °C and a 10 °C/min heating rate.

Differential scanning calorimetry (DSC) was performed using a NETZSCH 204 F1 Phoenix calorimeter (Netzsch Group, Selb, Germany). Samples of 5 ± 0.2 mg were cut from each granulate and placed in an aluminum crucible with a punctured lid. The measurements were performed under nitrogen in the 20–200 °C temperature range and at a 10 °C/min heating rate.

Heat distortion temperature (HDT) tests were carried out on specimens with dimensions corresponding to the flexural beam (4 × 10 × 80 mm), and the test was carried out by PN-EN ISO 75, HDT-A (1.8 MPa) [67] using Instron CEAST HV3 apparatus (Instron, Norwood, MA, USA). For all measurement series, three measurements were taken, and the result was averaged.

The effect of the modifier addition on the mass flow rate (MFI) was also determined. The measurements were made using an Instron plastometer (Instron, Norwood, MA, USA), model Ceast MF20, according to the applicable standard PN-EN ISO 1133 [68]. The measurement temperature was 210 ± 0.5 °C, while the piston loading was 2.16 kg.

The oscillatory shear rheological analysis was conducted at a temperature of 190 °C using an Anton Paar rotational rheometer (Anton Paar, Graz, Austria) equipped with parallel plate geometry, featuring a 25 mm diameter and a 1 mm gap. To identify the linear viscoelastic region (LVE) of all samples, initial dynamic strain amplitude sweep tests were performed in the 0.01–100% range at a constant frequency of 1 Hz, monitoring the storage modulus G′. Following this, dynamic frequency sweep measurements (0.1–100 Hz) were carried out for all samples at a strain value of 5%, as determined in the strain sweep test.

An evaluation of zero-shear viscosity (*η*_0_) was conducted through rheological measurements in oscillatory mode, utilizing calculations performed with the Rheoplus 32 v.3.62 software. The zero shear viscosity was determined by fitting the Carreau–Yasuda model to the experimental data. This model is represented by Equation (2):

(2) $$\eta \left(\dot{\gamma }\right)=\eta_{0}{\left[1+{\left(\lambda \dot{\gamma }\right)}^{a}\right]}^{\frac{n-1}{a}}$$

where

*η*_0_—zero-shear viscosity;

*n*—represents the power-law coefficient;

*a*—the adjustable exponent (2 for the simple Carreau model);

$\dot{\gamma }$—the shear rate, and *λ* is the characteristic time (further denoted as *λ_C-Y_*) [69].

Contact angle analyses were conducted at room temperature and atmospheric pressure using the sessile drop technique. A Krüss DSA 1 goniometer (Krüss Optronic GmbH, Hamburg, Germany) was utilized. To ensure accuracy, three distinct measurements were performed on each sample with a 5 µL water drop, to minimize the impact of surface nonuniformity. The outcome was determined by averaging the results obtained.

### 2.3. Synthesis Procedure of the Difunctional Organosilicon Derivatives

**Scheme 1:**
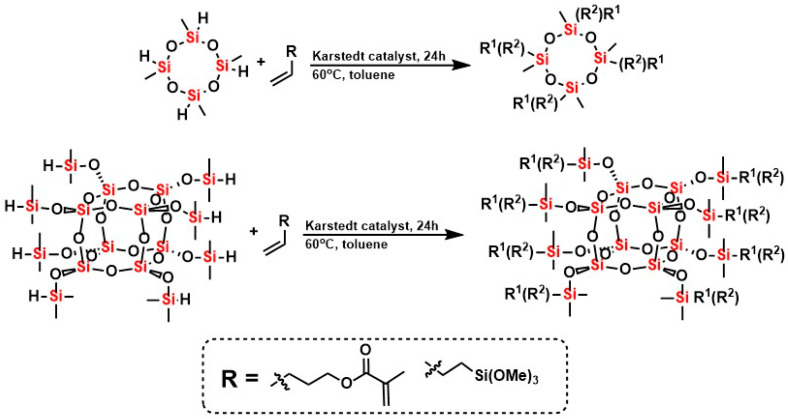
Hydrosilylation of olefins with octascpehrosilicate and cyclosilxoane.

The synthesis of OSS derivatives was performed corresponding to our previous work [70]. A total of 20 g of octaspherosilicate (OSS) or cyclosiloxane (CS) was loaded in a 500 mL three-neck, round-bottom flask, then 250 mL of toluene, and the mixture of olefin 1 and olefin 2 in the appropriate molar ratio, was added (see Tables S1 and S2). Then, Karstedt’s catalyst was added. The process was carried out under reflux for 24–48 h and heated to 60 °C. The reaction progress was monitored by FT-IR until the disappearance of the Si-H group bands (observed at 2141 cm^−1^ and 889 cm^−1^, corresponding to stretching and bending vibrations, respectively) (Scheme 1). All products exhibited a conversion above ~99% (indicated by the disappearance of the Si-H signal at 4.73 ppm), with an isolated yield of <95%.

Subsequently, the solvent was evaporated under a vacuum to dryness to obtain an analytically pure sample. The structure, conversion, and purity were verified using ^1^H, ^13^C, and ^29^Si NMR analysis (Supplementary File).

### 2.4. Technology Procedure for PLA Modification

PLA-based materials were obtained in a two-step process. Firstly, the masterbatches with 15 wt% of an organosilicon modifier were prepared using a two-roll laboratory mill ZAMAK MERCATOR WG 150/280 (Zamak Mercator, Skawina, Poland). PLA was heated to 215 °C until molten, then an appropriate portion of modifier was added, and the mixture was homogenized for 15 min. Then, the obtained material was granulated using a SHINI SG-1417-CE (Shini Plastics Technologies, Taichug, Taiwan) and dried at 60 °C/24 h.

An injection molding process achieved the final samples with the following modifier content: 5 wt%, 2.5 wt%, and 1 wt% in PLA matrix. Neat PLA granules were mixed with the masterbatch containing 15 wt% additive in a proper weight ratio and then formed into the shape of tested samples in agreement with PN-EN ISO 20753:2019-01 [71] (Figure S1). Figure S2 presents representative samples obtained for all series of modified PLA. Forming process parameter summaries are listed in Table S3.

## 3. Results and Discussion

### 3.1. Mechanical Properties Evaluation

#### 3.1.1. Impact Resistance

**Figure 1:**
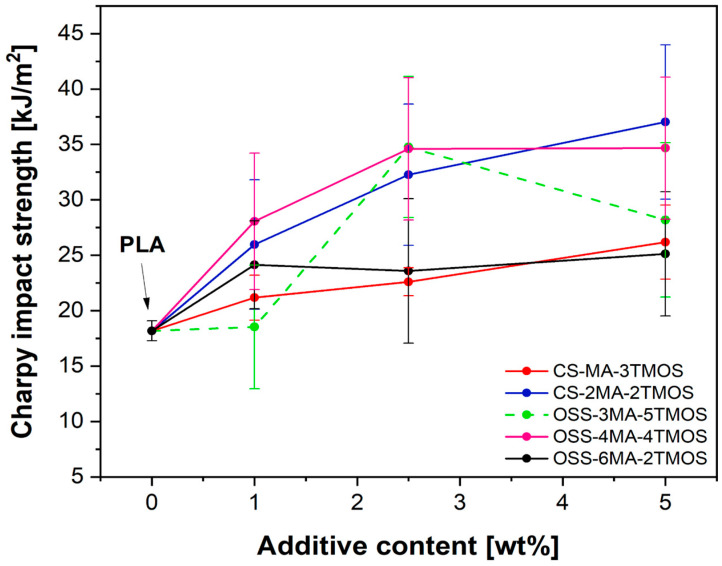
Relationship between the impact resistance of PLA and organosilicon additives concentration.

Charpy impact testing was carried out to determine the ability of the fabricated composites to absorb energy under dynamic loading conditions. The results obtained from the tests carried out are shown in Figure 1.

Neat PLA 2003D is classified as a polymer prone to brittle fracture [72,73], which is a significant limitation of its applicability and is considered a critical parameter to improve objects made from this material. Its impact strength was determined experimentally to be 18.18 ± 0.90 kJ/m^2^. Interestingly, the application of just 1 wt% modifier increases impact resistance by at least 15% for PLA modified with CS-MA-3TMOS and 55% for PLA with OSS-4MA-4TMOS. The observed enhancement in impact strength in relation to increasing concentrations of the modifier indicates a clear trend, with one exception noted for the PLA formulated with OSS-3MA-5TMOS. This anomaly may stem from the hydrolysis of the TMOS functional groups, as discussed in Section 3.6. A proportional increase in impact strength is evident with rising levels of CS-MA-3TMOS and CS-2MA-2TMOS in PLA, while the other additives show a slight decline at a 5% modifier content, though this is still considered within the range of measurement errors. The PLA/5%CS-2MA-2TMOS composite demonstrated the highest noted value with a remarkable 104% increase compared to neat PLA. Furthermore, the PLA/5%OSS-4MA-4TMOS featured a significant value, showing a 91% increase over neat PLA. Both PLA-based compositions contain organosilicon modifiers, in which the ratio of functional groups is 1:1. Compounds containing methacrylate groups are well known for their ability to undergo spontaneous thermally induced homopolymerization [74], which was confirmed by DSC analysis of selected organosilicon additives described in our earlier article [70]. During processing, the polymerization of the methacrylate groups present in the modifiers used results in the formation of interpenetrating structures between the organosilicon modifier particles and PLA. The use of simple silanes as coupling agents to improve adhesion between phases in various polymer systems has been widely reported in the literature [75,76]. The presence of trimethoxysilyl groups in the compounds used determines their uniform dispersion in PLA and good adhesion to the polymer matrix. This ensures cohesion between the phases of the composite, limiting potential nucleation sites for cracking, thus increasing their resistance to brittle fracture. The incorporation of rigid, spherical organosilicon-based particles—such as spherosilicates and tetracyclosiloxanes—demonstrates a marked improvement in the impact performance of PLA. These additives effectively dissipate mechanical energy and hinder crack propagation, leading to enhanced impact strength without compromising the inherent stiffness of the matrix. Compared to conventional plasticizers, particularly phthalate-based compounds, organosilicon additives offer distinct advantages related to their physicochemical stability. Notably, their low volatility and negligible tendency to migrate from the polymer bulk contribute to greater long-term material integrity. Furthermore, the incorporation of organosilicon compounds enhances the free volume among PLA macromolecules, which in turn influences the mobility of PLA chains. This process leads to plasticization, thereby improving the impact resistance of the material. In the context of the sustainable use of PLA products, the significant improvement in impact strength (~45% over PLA) obtained after already applying 1 wt% organosilicon additive is promising for future industrial applications.

#### 3.1.2. Static Tensile and Flexural Test Results

To comprehensively characterize the effects of spherosilicates and tetracyclosiloxanes on the mechanical properties of PLA, further tests of injected molded samples were carried out under static loading conditions—a tensile and a three-point bending test, comparison of the obtained result of flexural strength (Rf), flexural modulus (Ef), tensile strength (Rm), elastic modulus (Ey), and elongation at break (ε) is summarized in Table 1.

As the growth of modifiers loading in PLA, a slight decrease in Rf and Rm of composites was observed. The maximum difference in the Rf value and Rm value between the reference sample and PLA after modification was respectively ~8% (PLA/5%OSS-6MA-2TMOS) and 11% (PLA/OSS-4MA-4TMOS). The differences are caused by softening PLA segments in the presence of organosilicon additives, which are responsible for expanding the free volumes in the material [77]. A similar effect was observed by Dintcheva et al. in POSS-filled polystyrene nanocomposites [78]. The drop in mechanical properties by plastification of polylactide was also reported by Chaos et al. [79]. Moreover, organosilicon additives incorporated in PLA can lead to the accelerated formation of local discontinuities between PLA chains under tension and their expansion, consequently resulting in a marginal reduction in the strength of the polymer matrix.

The observed enhancement in the Ef modulus of the tested materials can be attributed to the presence of structures created by polymerized methacrylate groups derived from the utilized compounds in the PLA matrix. Microscopic structures created by cyclosiloxanes and OSS additives within PLA play a vital role in constraining local deformations and absorbing mechanical energy near the surface. These structures, spread between PLA macromolecules, experience both compression and tension, effectively distributing stress and limiting deformation. Consequently, this results in enhanced bending stiffness. At the same time, Young’s modulus decreases due to the formation of regions with varying stiffness, leading to an uneven stress distribution throughout the material under tension [80]. In all cases, tetracyclosiloxanes and OSS additives lead to a decrease in the composites’ Young’s modulus value.

Elongation at break of all modified samples shows a slight variation between the concentrations of each additive. For most of the samples, the elongation value is reduced compared to the reference due to the heterogeneities present in the material.

### 3.2. Microstructure Evaluation

#### 3.2.1. Distribution of Additives in PLA Composites—SEM-EDS Analysis

The compatibility between the modifier and PLA in PLA/CS and PLA/OSS systems was evaluated using SEM with energy dispersive X-ray spectroscopy. SEM-EDS maps illustrating the distribution of silicon (Si) are presented in Figure S3. Si dispersion maps were chosen as they indicate the presence of the modifier. However, it is worth noting that carbon (C) and oxygen (O) were also detected in all tested materials. The sample containing OSS-3MA-5TMOS has the highest concentration of silicon, which is reflected in additive chemical structure, and no larger agglomerates are observed. However, in the remaining samples, the analyzed areas exhibit varying concentrations of Si elements alongside empty spaces. Si is quite uniformly distributed at the micro level but tends to agglomerate due to the increase in the MA group in additive particles. Within the OSS-6MA-2TMOS sample, Si detection was the weakest and, to the greatest extent, uneven, with areas of higher density of the tested element. Additionally, notable agglomerates are evident in this sample, which correspond to the weakest Charpy impact strength results.

#### 3.2.2. A Fractography Study of the Breakthrough Surface After Dynamic Tension—Optical Microscopy (OM) and SEM Observations

**Figure 2:**
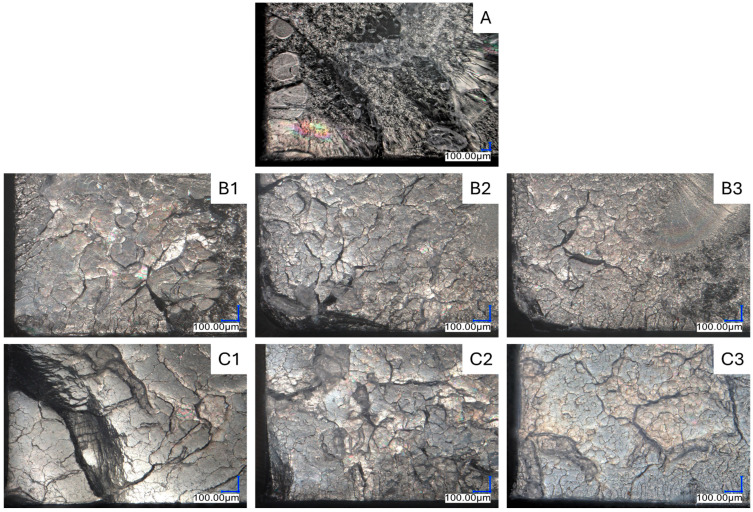

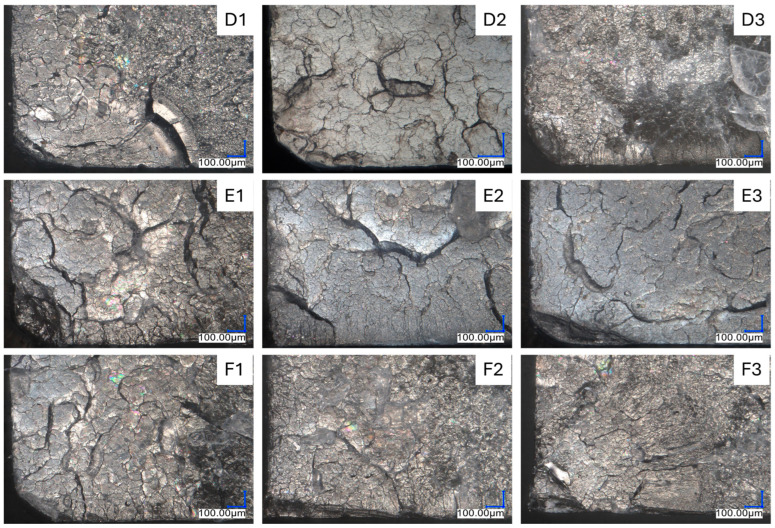
Optical microscopy pictures of the breakthrough surface after impact test: (**A**)—PLA, (**B1**–**B3**)—PLA/CS-MA-3TMOS, (**C1**–**C3**)—PLA/CS-2MA-2TMOS, (**D1**–**D3**)—PLA/OSS-3MA-5TMOS, (**E1**–**E3**)—PLA/OSS-4MA-4TMOS, (**F1**–**F3**)—PLA/OSS-6MA-2TMOS. For samples (**B1**–**F3**), 1 represents 1.0% additive loading, 2–2.5%, and 3–5.0%.

The microstructure images of modified PLA and the reference cross-section after the dynamic impact test are presented in Figure 2, with visible cracking areas. The distinctive attributes of brittle fracture are evident in the PLA reference, whereas the modified PLA exhibits characteristics akin to trans-crystalline-ductile behavior. The organosilicon compounds used to modify PLA have a dual function in influencing its plasticization and increasing the crystallinity degree of semicrystalline PLA (at 30–40% crystalline phase share). The primary failure mechanism observed in the studied materials is trans-crystalline fractures through crystalline regions with a higher ordering of PLA macromolecules characterized by a granular structure. At the same time, the modified materials show increased ductility in amorphous areas with a random distribution of polymer chains. When comparing the effect of additive concentration, a transition from a more brittle structure for lower modifier content to a more ductile structure with smoothed and rounded edges. A reduced number of granular areas with characteristic sharp and deep cracks is visible. A cohesion increases in the material structure after failure is also observed. The fractures of the samples containing OSS-3MA-5TMOS (Figure 2(D1–D3)) and OSS-6MA-2TMOS (Figure 2(F1–F3)) deviate from the general trend seen for the other materials, which corresponds to the results of the impact tests (Section 3.1). The presence of 2.5 wt% of the modifier OSS-3MA-5TMOS in the PLA structure results in its plasticization, but a further increase in the additive leads to a significant decrease in its impact resistance, resulting in a brittle fracture with significant granularity. The formation of modifier agglomerates in PLA is also observed. Similarly, the OSS-6MA-2TMOS additive, due to its high content of methacrylate groups, undergoes agglomeration in the polymer matrix, which, despite the partial strengthening of PLA, also constitutes internal notches.

**Figure 3:**
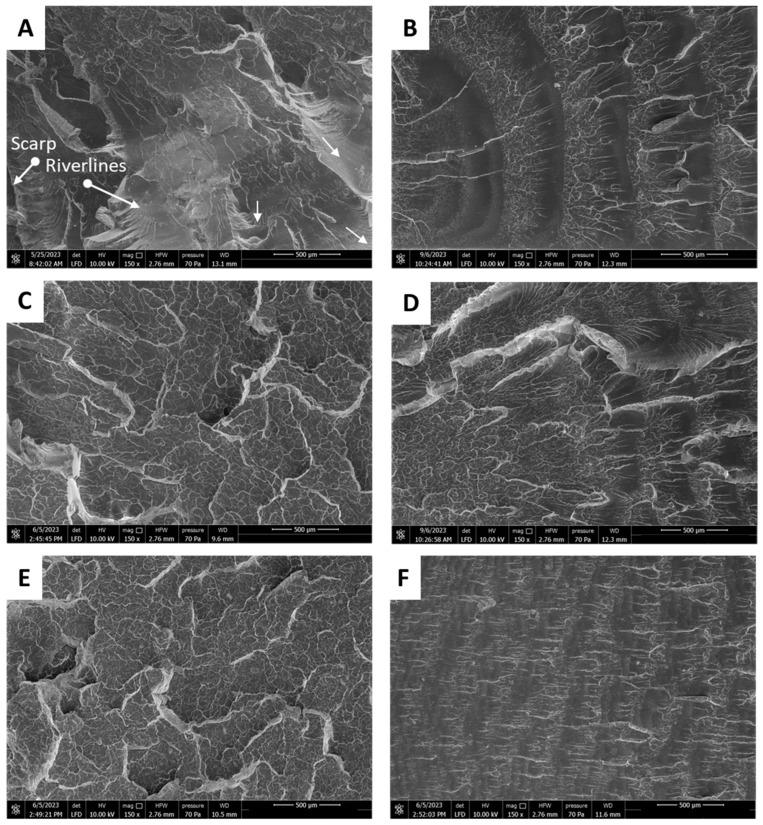
Impact of modifier used on PLA fracture mechanics during impact—SEM microscopy images of samples with 5 wt% additive: (**A**)—PLA, (**B**)—PLA/CS-MA-3TMOS, (**C**)—PLA/CS-2MA-2TMOS, (**D**)—PLA/OSS-3MA-5TMOS, (**E**)—PLA/OSS-4MA-4TMOS, (**F**)—PLA/OSS-6MA-2TMOS.

To conduct a more in-depth analysis of the modified PLA fracture behavior, we have selected samples with the highest additive concentration for further SEM microscopy examination. The key aspects of polymer deterioration are complex, but the mechanisms observed in both the initiation and propagation zones are widely recognized and firmly established in the field. Numerous studies have been conducted to investigate these mechanisms [81,82,83]. The initiation of the fracture morphology starts with crazing by the microvoids and the formation of the fibrils. While crazes initially absorb energy, their growth leads to full material failure. As an effect of damage propagation, the textured microflow is observed (Figure 3). White arrows in Figure 3A indicate directions of the crack propagation zone. The accumulation of multiple cracks in the polymer matrix leads to the formation of characteristic surface features such as scarps and river lines. SEM images of the breakthroughs, in addition to elemental SEM-EDS analysis, provide a qualitative assessment of the good mixing of the organosilicon additives used with the polylactide. PLA breakthroughs after the incorporation of organosilicon additives to the polymer matrix demonstrate a structure associated with ductile behavior. Compared to the reference PLA with a relatively smooth breakthrough surface, this area resembles a thread-like structure resulting from plastic deformation. Nanoparticles of the modifiers are used to act as barriers that slow down fracturing and increase strength through local deformation.

### 3.3. X-Ray Diffraction (XRD) Analysis Results

**Figure 4:**
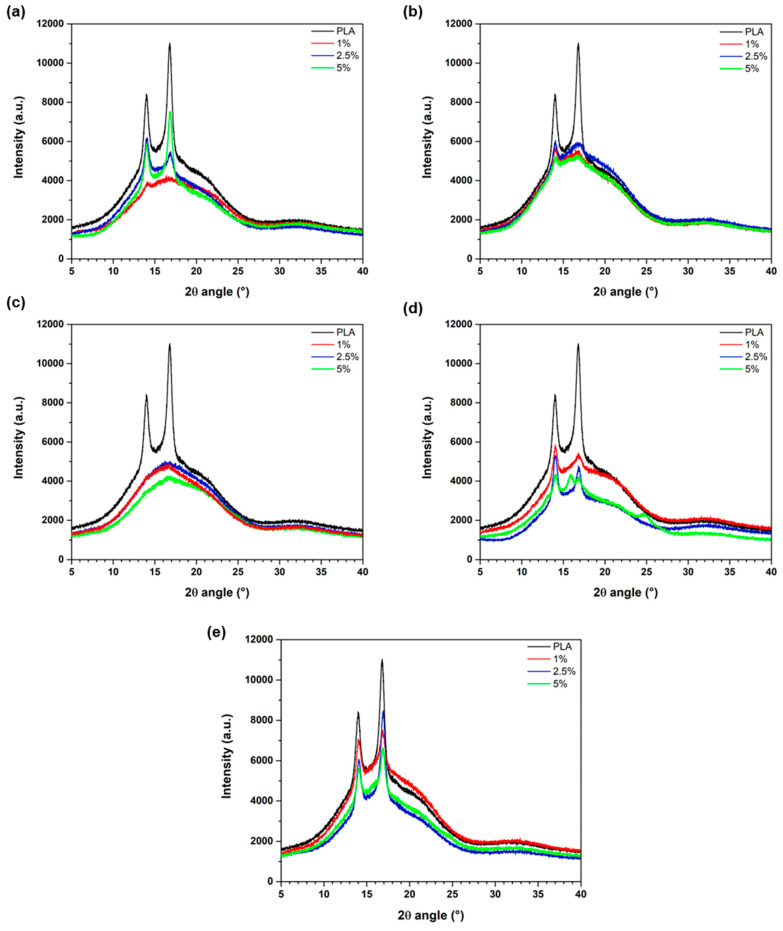
XRD patterns of PLA and PLA modified samples with (**a**) CS-MA-3TMOS, (**b**) CS-2MA-2TMOS, (**c**) OSS-3MA-5TMOS, (**d**) OSS-4MA-4TMOS, (**e**) OSS-6MA-2TMOS.

XRD analysis was carried out to trace the effects of functional cyclosiloxanes and OSS and their concentrations on changes in the crystalline structure of the PLA. Changes in PLA structure were tracked by performing surface analyses of injection-molded test bars. XRD patterns of modified PLA samples are shown in Figure 4. The diffractograms reflect the semicrystalline structure of the polylactide formed under standard processing conditions (relatively rapid cooling processes). The diffractograms are dominated by an amorphous ‘halo’ originating from areas of lower ordering of PLA macromolecules. Also present are reflections at 14° and 17° 2θ originating from orthorhombic crystals of the α-PLA crystalline phase [84,85]. Additional reflections at approximately 13°, 16°, and 25° 2θ are observed on the diffractograms of PLA modified with 5% OSS-4MA-4TMOS. Based on the data provided by Di Lorenzo and Androsch, the presence of a reflection at a flare angle of 2θ~25° allows the identification of the presence of less ordered α’-PLA metastable crystals in the obtained structure, besides the α-PLA phase and the amorphous part [86], which is also indicated by the results of the DSC analysis, to which more attention is devoted later in this work. The effect of the concentration of additives used is more complex and develops differently for individual polymer compositions. For materials containing the OSS-3MA-5TMOS additive, the amorphous background covers the reflections coming from the crystalline phase.

Analysis of the crystallinity degree of PLA and modified samples with the addition of functional organosilicone modifiers showed clear differences between individual samples (Table 2). For both CS-MA-3TMOS and CS-2MA-2TMOS modifiers, a moderate increase in crystallinity was observed with increasing concentration—from 25.2% to 31.7% and from 28.4% to 29.9%, respectively. The OSS-6MA-2TMOS modifier showed a significant effect on crystallization, which at 5% concentration reached the highest degree of crystallinity at the level of 42.3%. Similarly, OSS-4MA-4TMOS showed a change in crystallinity in relation to the change in concentration (from 31.7% to 38.2%). The OSS-3MA-5TMOS modifier had the lowest value among the tested samples (maximum value of 27.3%). The observed differences in crystallinity have a significant impact on the mechanical and processing properties of the materials, and a higher degree of structural order increases fracture toughness and materials’ thermal stability. The observed differences in crystallinity affect both the mechanical and processing properties of the materials; a higher degree of structural ordering increases the fracture toughness and thermal stability of the materials. However, the degree of crystallinity of PLA after modification is, for most samples, lower or close to the X_c_ value for neat PLA, which is related to the plasticizing properties of the organosilicon compounds described in the paper.

### 3.4. Thermal Analysis Results

#### 3.4.1. Differential Scanning Calorimetry (DSC) and Thermogravimetric Analysis (TGA)

**Figure 5:**
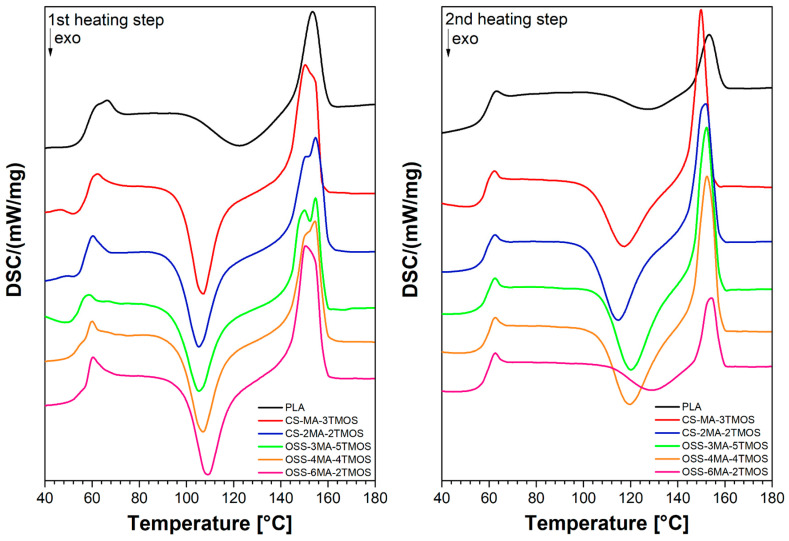
DSC curves of samples with 5 wt% additives.

In all samples, three typical events of semicrystalline polymer glass transition, cold crystallization, and melting phenomena were observed for both the first and second heating steps (Figures S4 and S5). Selected DSC curves obtained for 5 wt% additive are shown in Figure 5. It is clear that there is a distinct difference in the enthalpy of specific events on the DSC curves between the first and second cycles. In the majority of samples, the division of the melting peaks is prominently observed during the first heating cycle (for 1%OSS-3MA-5TMOS in the second heating too). The values corresponding to the split peak maxima observed in the melting temperature are designated as Tm1 and Tm2, respectively. Several mechanisms have been proposed to explain the dual melting peaks observed in PLA. Possible origins of the complex melting phenomenon are as follows: polymorphism (coexistence of multiple crystalline forms), variations in lamellar morphology (thickness, perfection, or stability), melting–recrystallization–remelting during heating physical aging, or relaxation of the rigid amorphous phase, and molecular weight distribution effects [87,88]. According to Xu et. al, the melting–recrystallization mechanism can explain the presence of double melting peaks (two polymorphic phases formation α and α’), as the previous crystallization took place below 120 °C, resulting in the formation of less perfect crystals (α’) [89]. It is further interesting that the initial double melting peak observed in the first DSC heating cycle transforms into a single sharp peak during reheating in the second DSC cycle, which is since α′ can recrystallize to the more stable α form. This transition reflects increased crystalline perfection and thermal stability of the material [90]. On the one hand, the melting–recrystallization model proposes that the initial endothermic event is due to the melting of imperfect lamellae (α’), which is followed by recrystallization into more stable structures (α) that melt at higher temperatures [91]. Conversely, the double lamellar thickness model explains the presence of dual endothermic peaks as a result of lamellae with varying thicknesses—thinner crystals melting at lower temperatures and thicker ones at higher temperatures. Consequently, with continued heating, recrystallization takes place, leading to the formation of crystallites of diverse shapes and sizes. Smaller crystals tend to melt at a lower temperature, corresponding to the first peak, whereas larger, more stable crystallites melt at a higher temperature, represented by the second peak. The minimal decrease in glass transition temperature (Tg) can be attributed to a reduction in the molecular entanglement of PLA chains resulting from the relaxation of PLA macromolecules due to the widening free spaces occupied by CS and OSS additives (Table 3). Similar findings were reported by Sirin et al. when they added 1–10 wt% A-POSS, G-POSS, T-POSS, and O-POSS into PLA [15]. The cold crystallization temperature (Tcc) of PLA was depressed by CS and OSS particles, indicating the nucleation nature of additives [92]. The dropoff of Tcc in PLA/CS samples was more noticeable than in PLA/OSS, especially in high CS particle loading.

**Figure 6:**
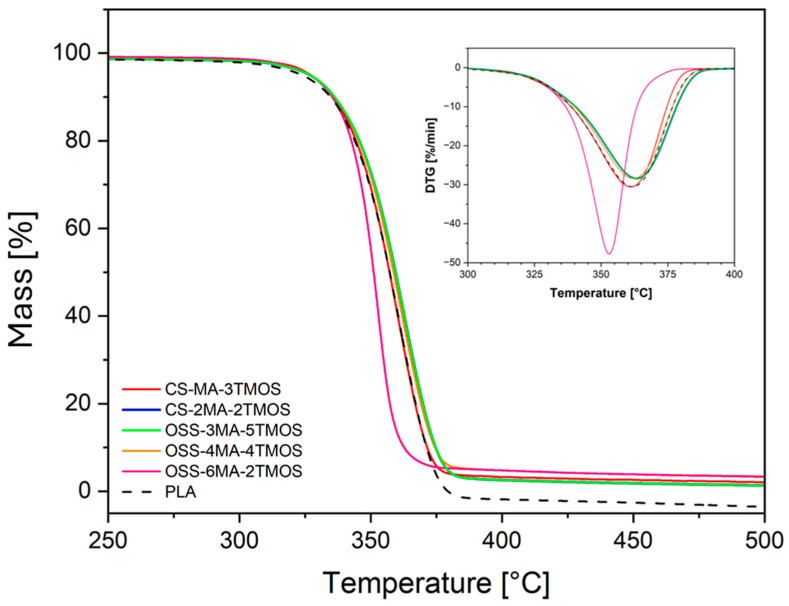
TGA and DTG curves of samples with 5 wt% additives.

The study of the effect of 5% of different types of CS and OSS modifiers on the thermal stability of PLA was carried out using TGA (Table 2). The obtained TGA curves and the derivative illustrating the DTG decomposition rate are presented in Figure 6. All tested samples, both neat PLA and modified, were characterized by a single-stage thermal decomposition process. The analysis showed that the initial decomposition temperature increased by 3–5 °C at 5% weight loss for the modified samples compared to unmodified PLA, for which T5% was 324.2 °C. The increase in this value indicates a slightly beneficial effect of the modifiers on the initial thermal stability of the material. No significant differences were observed in Tonset and Tmax for most of the modified samples, compared to neat PLA. The exception was the PLA sample with the addition of 5% OSS-6MA-2TMOS, for which a decrease in Tmax was observed. An interesting finding from comparing the results of this study with previous studies on the thermal stability of 3D-printed PLA modified by similar additives is that the thermal stability of injection-molded materials was lower than that of additively printed samples such as PLA/OSS-4MA-4TMOS and PLA/OSS-6MA-2TMOS. This phenomenon can be explained by differences in the thermal history of the materials associated with different processing techniques. In the 3D-printing process, PLA undergoes different thermal cycles and crystallization conditions compared to the injection molding process, which can lead to differences in the material structure and morphology [69].

#### 3.4.2. Heat Deflection Temperature (HDT)

The modified PLA is characterized by an increase in the HDT temperature (Table 2) relative to the reference PLA of approximately 10% (~5 °C). Cyclosiloxane-based additives, as their concentration increases, slightly increase the HDT temperature of PLA. However, HDT value decreases when CS additive loading rises, which can be attributed to a broader plasticizing effect with increasing additive concentration. In contrast, PLA samples modified with OSS additives do not show large differences in HDT depending on changes in additive concentration. Only for PLA/OSS-3MA-5TMOS, a slight difference is observed. In general, the increase, similar to the flexural stiffness modulus, in the HDT values of PLA after modification with organosilicon compounds containing methacrylate groups, can be explained by the stiffening of PLA chains through the modifier particles between PLA chains, previously discussed in the mechanical analysis Section.

### 3.5. Rheology

#### 3.5.1. Melt Flow Rate (MFR)

Melt flow behavior measurements were conducted as an easy method to assess the rheological characterization of PLA composites during processing under static conditions (see Table 4). The mass flow rate of neat PLA 2003D was experimentally determined according to standardized methods (210 °C, 2.16 kg) and was found to be approximately 6.5 g/10 min. The MFR results of the modified PLA presented show a significant increase with an increasing concentration of the additives used. Application of only 1 wt% of the organosilicon modifier to PLA allows for a substantial increase in the melt flow index value by as much as 46% for the composites with CS-based additives and more than 100% for the OSS. Broad literature reports indicate a significant effect of organosilicon compounds trimming the rheological properties of various polymeric materials [24,93]. Due to their lubrication properties, organosilicon compounds remarkably impact PLA’s rheology, even in small concentrations [20]. This phenomenon is caused by changes in polymer flow behavior, reducing intermolecular friction between polymer molecules and allowing them to move more freely past each other, thereby increasing chain mobility [94].

#### 3.5.2. Oscillatory Rheometry

The impact of CS- and OSS-based additives on the melt-rheological properties of PLA was assessed at 190 °C using oscillatory rheometry. Figure S6 illustrates the relationship between storage modulus and strain. The data indicate that the tested samples exhibit a Newtonian plateau in the 0.1–10% strain range. It is evident that all the samples display a smooth curve throughout the strain range, without a distinct onset point for non-linear viscoelastic behavior. In the case of PLA with OSS-4MA-4TMOS and OSS-6MA-2TMOS samples, a reduction in G′ is noted for lower additive concentrations (1% and 2.5%), while an increase is observed for samples containing 5 wt% of the modifier. This phenomenon may be attributed to modifier migration or phase separation [95]. The lowering G′ may occur because of relatively low content or the absence of strain-sensitive rigid network-like structures between additives and progressive plasticization of the PLA matrix [96]. The curves for the other materials follow virtually the same pattern.

**Figure 7:**
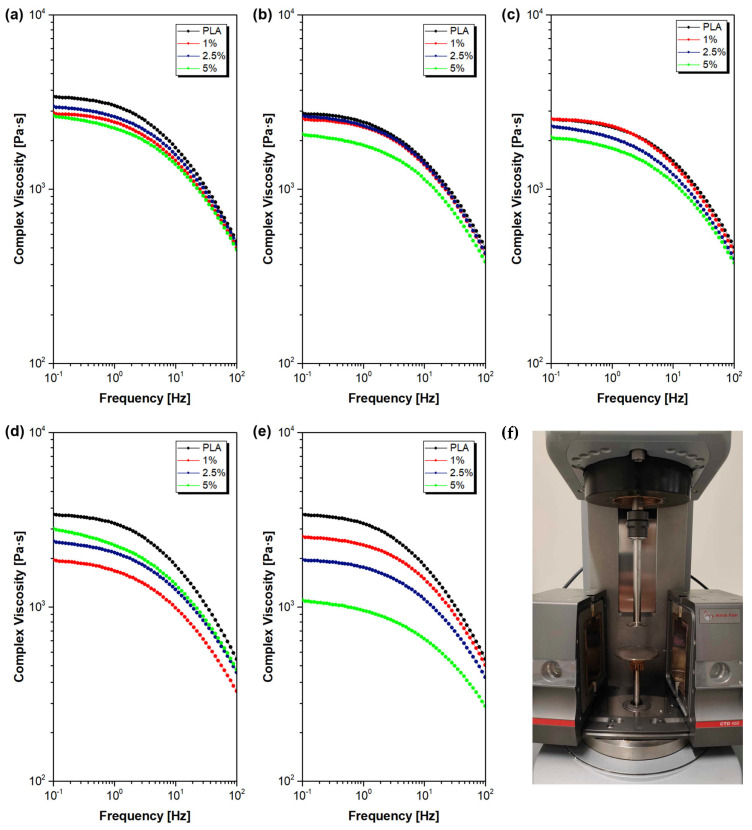
Complex viscosity curves of PLA and its modified samples—dependence of modifier concentration: (**a**) OSS-3MA-5TMOS, (**b**) OSS-4MA-4TMOS, (**c**) OSS-6MA-2TMOS, (**d**) CS-MA-3TMOS, (**e**) CS-2MA-2TMOS, (**f**) measuring setup.

The complex viscosity determined by the frequency-sweep test (Figure 7) demonstrates a notable dependency on additive concentrations. As the loading of the organosilicon modifier increases, there is a gradual decrease in complex viscosity, except for samples with OSS-4MA-4TMOS. Viscosity remains constant at lower frequencies, suggesting no significant change with frequency. However, at higher frequencies (>1 Hz), a noticeable decrease in viscosity is observed, indicating shear-thinning behavior characteristic of non-Newtonian fluids. This behavior is typical of pseudoplastic materials, like most polymers. It can be attributed to the disentanglement and increase in free volumes in the melt state due to the presence of modifier particles, leading to a reduction in viscous resistance [97]. Romo-Uribe observed that the addition of oligomeric silsesquioxane–styrene copolymers to polystyrene indicates a molecular lubrication effect, producing chain disentanglement and thus reducing the melt viscosity [98]. A significant deviation from the curve for neat PLA is visible for PLA/organosilicon samples at a 5 wt% modifier loading, indicating plasticization of the material when smaller quantities of organosilicon compounds may act more like lubricating agents [93]. Only the PLA samples with OSS-4MA-4TMOS showed increased complex viscosity dependent on modifier concentration, but still much lower than the reference. This may be due to the aggregation of the additive and the blocking of PLA chain motion.

Based on Figures S7 and S8, it is evident that for each material—both neat PLA and those containing the additives—the loss modulus G″ exceeds the storage modulus G′ across the entire frequency range. This suggests that the behavior of the tested materials aligns more closely with viscous rather than elastic within the depicted frequency range. The loss and storage modulus of PLA/organosilicon samples show lower values across the entire frequency range compared to neat PLA. The change in modulus is directly proportional to the additive loading in PLA compositions, except for PLA/CS-MA-3TMOS samples, which demonstrate the opposite trend. Unlike layered nanostructures or carbon nanotubes, organosilicon additives have a low aspect ratio. According to Yazdaninia et al., even when the POSS content is as high as 20 wt%, interconnection structures may not form. Therefore, the observed change in rheological behavior is mainly affected by some additive organization into small agglomerates [93]. Changes in the crossover point for all samples are collected in Table 3. The sample with 5% CS-2MA-2TMOS does not present the G′ = G″ point in the considered frequency range. The relaxation time depends on both how stiff the material is (crossover value) and how quickly it responds to deformation (crossover frequency), so the results presented are quite complex and dependent on both additive concentration and type [99]. The higher frequency values of the G′ = G″ point suggest a shorter relaxation time for most of the samples at higher additive concentrations, but lower concentrations (1%) may have longer relaxation times in some cases (e.g., CS-MA-3TMOS and OSS-6MA-2TMOS) [100].

**Figure 8:**
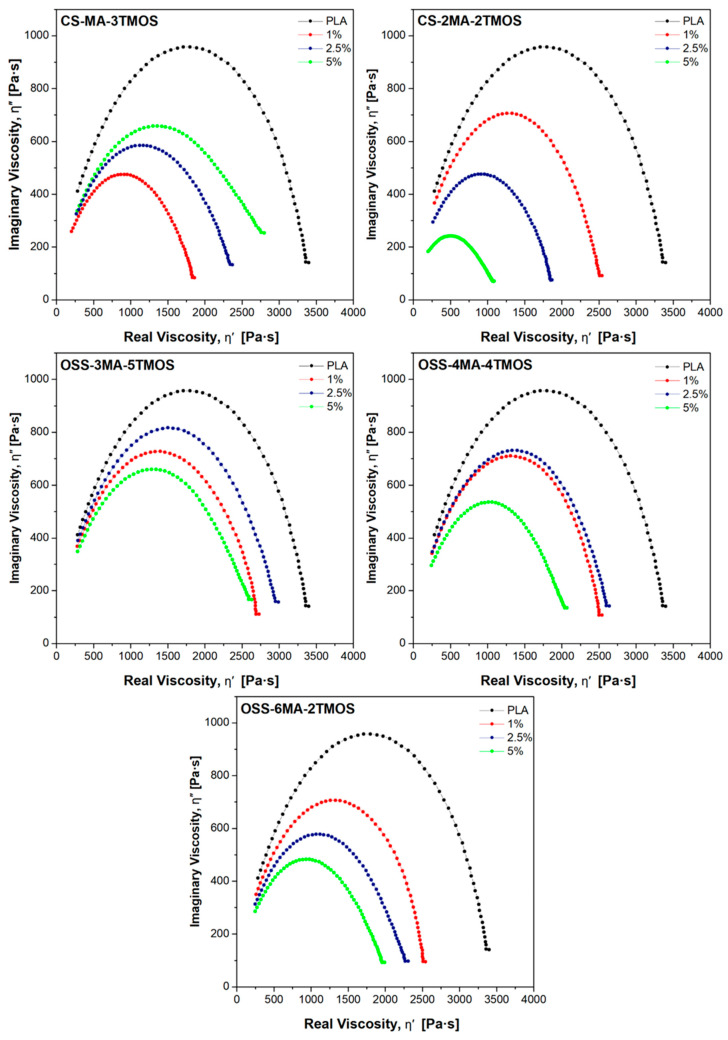
Cole–Cole plots of PLA and its modified samples.

**Figure 9:**
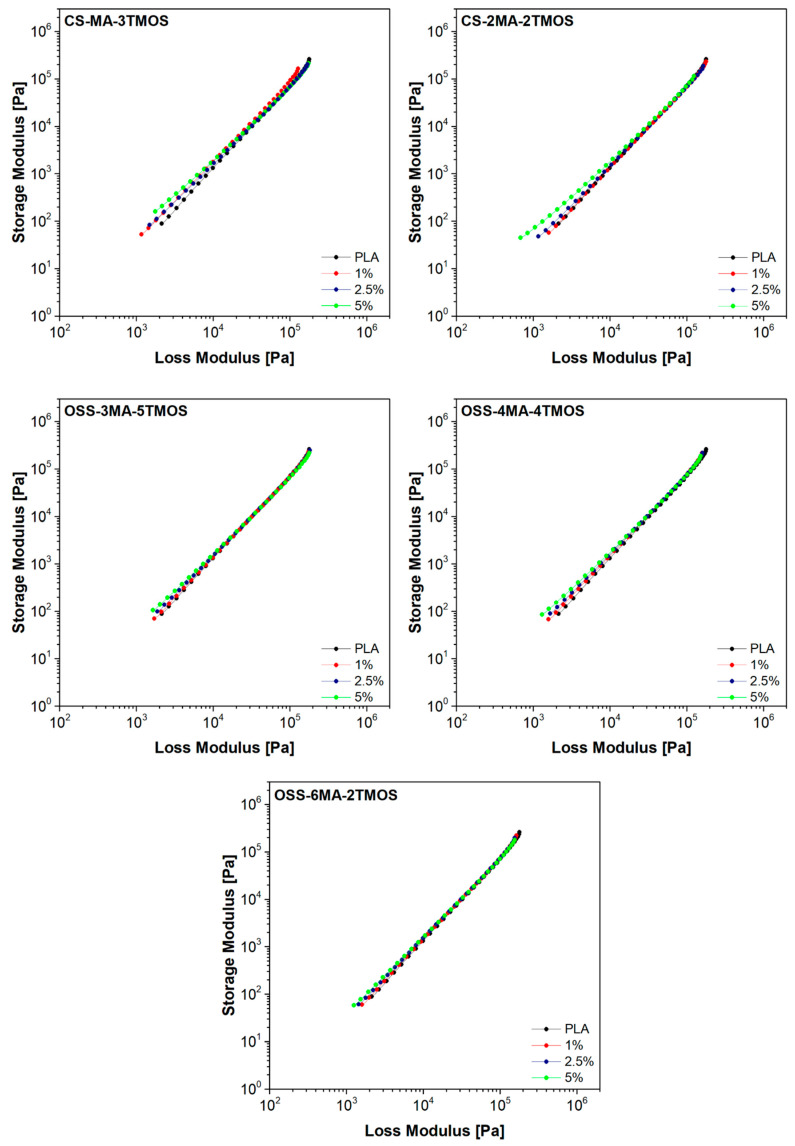
Han plots of PLA and its modified samples.

To identify the presence of structural changes, miscibility of PLA systems components, and their compatibility in the molten state the Cole–Cole, modified Cole–Cole (Han plot), and van Gurp–Palmen (vGP plot) charts were used as useful tools in rheological data interpretation [101,102,103,104]. The Cole–Cole plots of PLA and its modified samples are presented in Figure 8. The smooth, semicircle-shaped plotted curves suggest high compatibility between PLA and the additives utilized. This indicates that the obtained samples exhibit good homogeneity in the molten state, with finely dispersed additives. The Cole–Cole curves of the modified PLA samples demonstrate the lubricating effect of the additives, consistent with the findings reported by Zhou et al. for polypropylene-containing POSS materials. This is evident by the observed downward shift in the position of the Cole–Cole curves compared to PLA [105]. The Han plots allow us to eliminate the effect of temperature and frequency on the properties of viscoelastic polymers. The curves logG′-logG″ (Figure 9) indicate a slight deviation from the linear trend at a higher modifier content. As has been shown, no phase separation occurs for the obtained samples. The graphs show that the curves closely overlap, with a small sample deviation of 5%, consistent with the viscosity values. Furthermore, this deviation suggests a genuine change in the mobility of the PLA macromolecules in the presence of the additive, indicating its plasticizing effect. The Han slope value is lower for samples modified with CS, supporting the conclusion that these compounds are better plasticizers than OSS. This may be related to the lower molecular weight of these plasticizers [106,107].

**Figure 10:**
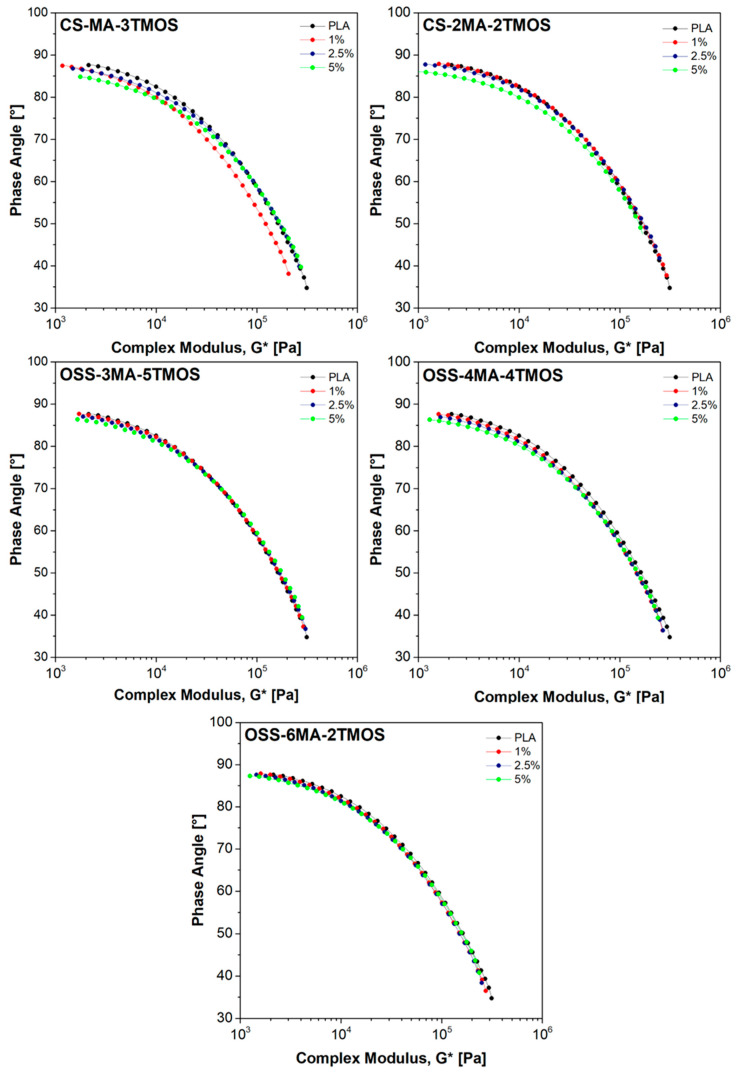
vGP plots of PLA and its modified samples.

Van Gurp–Palmen analyses were conducted for PLA and PLA/organosilicon samples by examining the correlation between the phase angle (δ) and complex modulus (G*), as illustrated in Figure 10. The phase angle indicates the phase difference between applied strain and measured stress. It approaches 0° for materials exhibiting purely elastic behavior (indicating strain and stress are in phase) and 90° for viscous materials (indicating two waves out of phase) [108]. The van Gurp–Palmen diagram offers a more intuitive representation of material behavior at different frequencies than direct G′ and G″ diagrams [109]. A high value of *δ* at low G* indicates the dominance of viscous properties. The plotted curves confirm good compatibility of PLA and modifiers without phase transitions or the formation of neat-like structures providing significant changes in polylactide microstructure, which has been observed by Wang for nanosilica-compatibilized polymer blends and Wu in biodegradable polylactide/carbon nanotube composites [109,110]. The lack of maximum on the curves is presented, and *δ* decreases continuously with increasing complex modulus. The vGP analysis shows that the additives are well spread out in the PLA material, indicating no significant deviation from the standard polymer curve without a clearly defined percolation threshold, in contrast to changes observed by Ivanova and Kotsilkova in PLA nanocomposites [111].

In the literature, the analysis of the characteristic relaxation times was performed based on an oscillation test using various relaxation mathematical models. The characteristic relaxation time *λ* was evaluated based on two complementary approaches corresponding to different rheological models. Considering the linear viscoelastic single-mode Maxwell model, which was proposed for a description of the frequency dependence of the moduli, the angular frequency ω*_c_* at the crossover point corresponds to the longest relaxation time. *λ_M_*, which represents the time scale over which stress relaxes after a sudden deformation, was estimated from the crossover point G′ = G″ based on for Equation (3) [112]:

(3) $$\lambda_{M}=\eta_{0}J_{M}^{0}=\frac{1}{\omega_{c}}$$

where *λ_M_* relaxation time according to the Maxwell model, *η*_0_ is the zero-share viscosity, $J_{M}^{0}\;$is the linear steady-state recoverable compliance, and ω*_c_* is the angular frequency. Additionally, the values of characteristic relaxation time *λ_C-Y_* were calculated from Equation (1) related to the onset of non-Newtonian or shear thinning behavior [113,114].

Both the relaxation time derived from the Maxwell model *λ_M_* and that obtained from the Carreau–Yasuda model *λ_C-Y_* systematically decrease with increasing modifier concentration from 1% to 5%, which is typical for plasticizer molecules that penetrate the PLA matrix, increasing the free volume and spacing between polymer chains. As a result, the intermolecular interactions and entanglements are weakened, facilitating faster stress relaxation. This observation directly reflects the modification of the internal structure and viscoelastic behavior of the PLA matrix. The consistent reduction in zero-shear viscosity (*η*_0_) supports the interpretation of enhanced molecular flowability. Similar observations were reported by Angel Romo-Uribe [115] for POSSn-MMA and for PE/octaisobutyl-POSS composites [116].

### 3.6. Water Contact Angle (WCA)

The WCA measurements of PLA and PLA/organosilicon samples were conducted to assess their surface hydrophobic/hydrophilic properties for understanding how the PLA modification influences interaction with water, which may be crucial for its potential future applications. Pictures of droplets are presented in Figure S9. PLA generally belongs to materials with hygroscopic properties, which is why drying it before plastic processing is recommended. The introduction of organosilicon derivatives into PLA (WCA~78.83°) resulted in a slight increase in WCA toward more hydrophobic properties (hydrophobic materials WCA > 90°) [117]. The results presented in Table 5 show that an increase in additive concentration leads to higher WCA values. However, the significant dropoff in WCA PLA/OSS-3MA-5TMOS samples can be explained by the partial hydrolysis of trimethoxysilyl groups to hydroxyl groups induced by high temperatures in Equation (4).

(4) $$-Si{\left(OMe\right)}_{3}+3H_{2}O\to -Si{\left(OH\right)}_{3}+3MeOH$$

The presence of an excessive number of trimethoxysilyl groups can hinder hydrolysis due to interactions between the groups, which can form lattice structures that limit water access. Efficient hydrolysis requires free access of water to the trimethoxysilyl groups, which can be challenged in a dense polymer matrix. Conversely, too few trimethoxysilyl groups may result in insufficient active sites for hydrolysis, leading to negligible effects [118,119,120].

## 4. Conclusions

This study demonstrates the effectiveness of five organosilicon derivatives—based on tetracyclosiloxane (CS) and octaspherosilicate (OSS) cores functionalized with methacrylate (MA) and trimethoxysilyl (TMOS) groups—as performance enhancers for polylactide (PLA) via melt compounding.

Notably, the addition of 1 wt% organosilicon additive increases impact strength by ~45%, with PLA/5%CS-2MA-2TMOS achieving a remarkable 104% improvement. Mechanical characterization revealed concurrent improvements in flexural strength, tensile strength, strain at break, and stiffness—particularly flexural modulus—suggesting effective stress distribution and energy dissipation through nano- and microstructural interactions facilitated by reactive MA groups. Fractographic (OM/SEM) analysis supported this by showing ductile fracture behavior, with dispersed modifiers acting as crack-arresting domains.

Thermal analysis (DSC) revealed a plasticizing effect, reflected by a reduction in the glass transition temperature (Tg), alongside a nucleating effect that led to decreased cold crystallization temperature (Tcc), both indicative of enhanced polymer chain mobility due to reduced molecular entanglement of PLA macromolecules and facilitated crystallization. XRD analysis confirmed the dominance of the thermodynamically stable α crystalline phase, with a minor presence of α′ in select composites (PLA/5% OSS-4MA-4TMOS). Furthermore, the thermal and thermomechanical stability of PLA was improved, with HDT values reaching up to 57 °C.

The rheological analysis allowed us to elucidate the mechanisms behind the interaction of CS and OSS additives with PLA, highlighting their dominant lubricating, rather than a strong plasticizing effect—evidenced by reduced viscosity and increased melt flow rate—without severely compromising structural integrity. Compatibility and miscibility of finely dispersed additives in the PLA matrix were confirmed through Cole–Cole, van Gurp–Palmen, and Han plots, further supported by uniform silicon distribution in SEM-EDS mapping. Han diagrams indicated a real increase in the mobility of PLA macromolecules in the presence of organosilicon additives, underscoring their plasticizing effects. These rheological findings, along with viscoelastic data, were consistent with morphological observations. EDS mapping of silicon distribution confirmed excellent dispersion of the organosilicon modifiers in the polymer matrix, likely due to the presence of adjacent trimethoxysilyl groups.

Moreover, the incorporation of organosilicon derivatives into PLA slightly increased the water contact angle (WCA), imparting more hydrophobic characteristics to the composites.

The results confirm that melt blending of PLA with carefully designed organosilicon compounds offers a viable and scalable strategy for enhancing its performance. The ability to fine-tune the core architecture and functional group content of the modifiers enables precise control over the resultant material properties. Among the tested derivatives, CS-2MA-2TMOS and OSS-4MA-4TMOS demonstrated the most pronounced improvements, even at low loadings, underscoring their potential for advanced applications in sustainable polymer technologies for consumer goods. These PLA-based materials could have promising applications in the fabrication of automotive interior components, protective housing for consumer electronics, and lightweight sporting equipment—areas where a balance of durability and environmental performance is increasingly sought.

## 5. Patents

The results of this publication have been patented with Polish patent application No. P.447903 and No. P.447904.

## Figures and Tables

**Table 1:** Summary table of static tensile and three-point bending test results of PLA and its modified samples.

Sample Name	Modifier (wt%)	Rf (MPa)	Ef (MPa)	Rm (MPa)	ε (%)	Ey (MPa)
**PLA**	-	101.03 ± 0.28	3497.18 ± 13.00	66.11 ± 1.70	2.61 ± 0.07	3284.19 ± 52.65
**PLA/CS-MA-3TMOS**	1	100.27 ± 1.54	3690.38 ± 46.40	64.13 ± 1.25	2.59 ± 0.07	3201.63 ± 47.09
	2.5	98.85 ± 0.32	3661.71 ± 17.11	63.25 ± 0.77	2.55 ± 0.04	3173.26 ± 41.52
	5	95.01 ± 0.67	3609.20 ± 6.06	63.18 ± 1.32	2.73 ± 0.08	2971.66 ± 98.88
**PLA/CS-2MA-2TMOS**	1	99.20 ± 0.42	3697.11 ± 13.67	63.87 ± 0.55	2.53 ± 0.04	3241.24 ± 38.84
	2.5	95.35 ± 1.09	3648.86 ± 28.18	60.46 ± 1.25	2.38 ± 0.03	3230.36 ± 23.96
	5	93.34 ± 1.57	3615.38 ± 42.36	58.39 ± 1.19	2.44 ± 0.03	3109.54 ± 58.21
**PLA/OSS-3MA-5TMOS**	1	98.03 ± 0.77	3701.57 ± 25.84	61.09 ± 1.48	2.40 ± 0.05	3241.47 ± 20.23
	2.5	96.22 ± 0.32	3610.46 ± 23.86	62.61 ± 1.48	2.57 ± 0.13	3111.34 ± 80.10
	5	93.08 ± 1.26	3297.12 ± 30.5	60.55 ± 1.08	2.61 ± 0.12	3043.58 ± 112.90
**PLA/OSS-4MA-4TMOS**	1	97.01 ± 0.23	3442.67 ± 16.84	61.55 ± 0.7	2.36 ± 0.02	3322.9 ± 30.20
	2.5	96.36 ± 0.45	3638.49 ± 29.00	58.60 ± 1.43	2.39 ± 0.08	3236.02 ± 17.84
	5	95.21 ± 0.53	3575.26 ± 43.47	58.83 ± 1.17	2.57 ± 0.06	3119.93 ± 20.72
**PLA/OSS-6MA-2TMOS**	1	97.21 ± 0.54	3616.06 ± 96.63	62.11 ± 0.93	2.50 ± 0.05	3244.85 ± 39.27
	2.5	95.90 ± 0.76	3636.19 ± 12.97	60.72 ± 0.28	2.49 ± 0.05	3158.08 ± 54.62
	5	92.88 ± 0.88	3576.5 ± 24.50	59.14 ± 0.84	2.62 ± 0.11	3066.76 ± 37.99

**Table 2:** Degree of crystallinity of PLA and its modified samples.

	Modifier (wt%)	PLA	PLA/CS-MA-3TMOS	PLA/CS-2MA-2TMOS	PLA/OSS-3MA-5TMOS	PLA/OSS-4MA-4TMOS	PLA/OSS-6MA-2TMOS
**Crystallinity degree X_c_ (%)**	-	30.49	-	-	-	-	-
	1	-	25.2	28.4	21.7	31.7	36.3
	2.5	-	29.4	29.5	25.9	33.4	35.8
	5	-	31.7	29.9	27.3	38.2	42.3

**Table 3:** DSC and TGA results of PLA and its modified samples.

Sample Name	Modifier (wt%)	T_g_ (°C)	T_cc_ (°C)	T_m1/_T_m2_ (°C)	T_5%_ (°C)	T_onset_ (°C)	T_max_ (°C)	HDT (°C)
**PLA**	-	63.2	127.2	155.4	324.2	342.2	362.1	52.80 ± 0.15
**PLA/CS-MA-3TMOS**	1	62.8	129.2	153.8	-	-	-	57.50 ± 0.26
	2.5	62.7	119.4	151.9	-	-	-	56.80 ± 0.20
	5	61.8	116.9	150.1	327.4	341.8	361.2	54.70 ± 0.20
**PLA/CS-2MA-2TMOS**	1	62.6	121.2	152.3	-	-	-	57.50 ± 0.10
	2.5	61.8	113.2	150.7/155.5	-	-	-	56.83 ± 0.15
	5	62.4	114.5	151.3	327.0	343.1	363.3	54.93 ± 0.32
**PLA/OSS-3MA-5TMOS**	1	62.0	111.8	149.6/156.1	-	-	-	57.75 ± 0.15
	2.5	62.8	120.9	152.8	-	-	-	56.60 ± 0.10
	5	62.4	120.5	152.0	329.7	345.3	360.5	55.87 ± 0.21
**PLA/OSS-4MA-4TMOS**	1	63.2	122.6	153.2	-	-	-	56.90 ± 0.00
	2.5	62.7	121.2	152.7	-	-	-	57.23 ± 0.23
	5	62.7	119.9	152.6	327.2	342.5	362.1	56.57 ± 0.23
**PLA/OSS-6MA-2TMOS**	1	63.2	120.5	152.7	-	-	-	57.71 ± 0.28
	2.5	62.8	128.7	153.5	-	-	-	57.36 ± 0.05
	5	62.7	129.0	154.1	327.1	342.1	353.2	56.27 ± 0.06

**Table 4:** Rheological parameters of PLA and its modified samples.

Sample Name	Modifier [wt%]	ω_cross_ [rad/s]	G′ = G″ [kPa]	λ_M_ [ms]	λ_C-Y_ [ms]	η_0_ [Pa·s]	Han Plot Slope	MFR_(210 °C, 2.16 kg)_ [g/10 min]
**PLA**	-	212.42	148.24	4.71	19,12	3486.10	1.76	7.11 ± 0.03
**PLA/CS-MA-3TMOS**	1	324.19	111.22	3.08	10.07	3031.80	1.68	9.55 ± 0.01
	2.5	385.28	154.75	2.60	8.57	2483.20	1.63	9.50 ± 0.03
	5	369.71	158.48	2.70	4.46	1937.30	1.55	10.98 ± 0.05
**PLA/CS-2MA-2TMOS**	1	300.25	155.03	3.33	9.78	2590.10	1.73	9.58 ± 0.02
	2.5	480.21	158.16	2.08	8.85	1921.50	1.66	10.72 ± 0.08
	5	-	-	-	1.99	1147.90	1.52	13.45 ± 0.03
**PLA/OSS-3MA-5TMOS**	1	284.90	150.55	3.51	13.81	3074.00	1.72	10.59 ± 0.29
	2.5	260.91	153.52	3.83	11.41	2823.70	1.68	15.50 ± 0.91
	5	361.11	161.84	2.77	5.81	2748.50	1.61	16.30 ± 0.27
**PLA/OSS-4MA-4TMOS**	1	258.54	133.74	3.87	16.43	2582.10	1.72	10.84 ± 0.05
	2.5	255.36	134.86	3.92	12.17	2704.50	1.68	9.29 ± 0.03
	5	371.55	138.39	2.69	5.20	2137.60	1.60	13.23 ± 0.05
**PLA/OSS-6MA-2TMOS**	1	264.99	137.46	3.77	17.06	2593.70	1.74	10.27 ± 0.09
	2.5	332.49	138.07	3.01	6.06	2417.00	1.70	9.02 ± 0.07
	5	434.56	144.68	2.30	4.49	2090.50	1.65	13.16 ± 0.03

**Table 5:** WCA results of PLA and its modified samples.

	Modifier (wt%)	PLA	PLA/CS-MA-3TMOS	PLA/CS-2MA-2TMOS	PLA/OSS-3MA-5TMOS	PLA/OSS-4MA-4TMOS	PLA/OSS-6MA-2TMOS
**WCA (°)**	-	78.83 ± 0.96	-	-	-	-	-
	1	-	83.93 ± 1.55	88.76 ± 1.58	37.40 ± 1.30	77.57 ± 1.31	83.13 ± 1.93
	2.5	-	83.68 ± 0.78	89.07 ± 0.21	44.93 ± 1.32	76.65 ± 1.85	83.67 ± 2.40
	5	-	82.78 ± 1.05	92.33 ± 1.73	50.40 ± 1.56	84.13 ± 1.80	87.03 ± 2.34

## Data Availability

The original contributions presented in this study are included in the article and Supplementary Materials. Further inquiries can be directed to the corresponding author.

## Supplementary Materials

The following supporting information can be downloaded at: https://www.mdpi.com/article/10.3390/ma18122903/s1. Table S1: Structures of octaspherosilicate products obtained from the hydrosilylation reaction for polymer modification. Table S2: Structures of cyclosiloxane products obtained from the hydrosilylation reaction for polymer modification. Table S3: Injection molding parameters. Figure S1: Injection molding set up for polylactide specimen preparation. Figure S2: Influence of the modifier on appearance prepared samples—representative images of injection-molded specimens for tensile test (ISO type 1A). Figure S3: Organosilicone additives distribution in PLA matrix—SEM-EDS elemental mapping of Si on the breakthrough surface after impact detected for modified PLA with 5 wt% additive. Figure S4: DSC thermograms of PLA modified with cyclosiloxane additives. Figure S5: DSC thermograms of PLA modified with spherosilicate additives. Figure S6: Strain dependence of storage modulus for PLA and modified samples at 190 °C. Figure S7: Storage modulus (G′) vs. loss modulus (G″) frequency dependence at 190 °C of PLA and samples modified with cyclosiloxane additives. Figure S8: Storage modulus (G′) vs. loss modulus (G″) frequency dependence at 190 °C of PLA and samples modified with octaspherosilicate additives. Figure S9: The pictures of a water droplet applied on the surface. A—PLA, B—PLA/CS-MA-3TMOS, C—PLA/CS-2MA-2TMOS, D—PLA/OSS-3MA-5TMOS, E—PLA/OSS-4MA-4TMOS, F—PLA/OSS-6MA-2TMOS.
