# Supplementary material for: Dual-Functional Organosilicon Additives Containing Methacrylate and Trimethoxysilyl Groups Enhancing Impact Toughness of Polylactide (PLA): Structure–Property Relationship
Source: Materials (Basel). 2025 Jun 19;18(12):2903. doi: 10.3390/ma18122903 (PMC12195416; doi:10.3390/ma18122903)
Supplement: Supplementary file 1 [file materials-18-02903-s001.zip › materials-3644776-supplementary.pdf]

materials

MDPI

Article

Dual-Functional Organosilicon Additives Containing Methacrylate and Trimethoxysilyl Groups Enhancing Impact Toughness of Polylactide (PLA): Structure–Property Relationship

Julia Głowacka [1,2], Miłosz Frydrych [1], Eliza Romańczuk-Ruszuk [3], Yi Gao [4], Hui Zhou [4], Robert E. Przekop [1] and Bogna Sztorch [1,*]

1 Center for Advanced Technologies, Adam Mickiewicz University Poznan, Uniwersytetu Poznanskiego 10, 61-614 Poznan, Poland; julia.glowacka@amu.edu.pl (J.G.); frydrych@amu.edu.pl (M.F.); rprzekop@amu.edu.pl (R.E.P.)
2 Faculty of Chemistry, Adam Mickiewicz University Poznan, Uniwersytetu Poznanskiego 8, 61-614 Poznan, Poland
3 Institute of Biomedical Engineering, Faculty of Mechanical Engineering, Bialystok University of Technology, Wiejska 45C, 15-351 Bialystok, Poland; e.romanczuk@pb.edu.pl
4 Key Laboratory for Thermal Science and Power Engineering of Ministry of Education, Beijing Key Laboratory of CO_2 Utilization and Reduction Technology, Department of Energy and Power Engineering, Tsinghua University, Beijing 100084, China; 18gy@mail.tsinghua.edu.cn (Y.G.); huizhou@tsinghua.edu.cn (H.Z.)
* Correspondence: bogna.sztorch@amu.edu.pl

1. Chemical structures of the additives

Table S1. Structures of octaspherosilicate products obtained from the hydrosilylation reaction for polymer modification.

Modifier code	Chemical formula	TMOS:MA molar ratio	Name
OSS-3MA-5TMOS	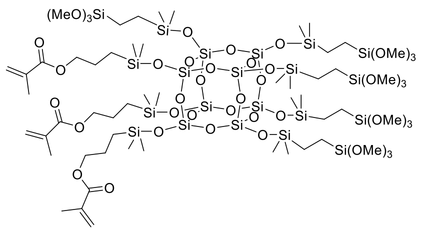	3:5	1,3,5,7,9,11,13,15-tri((propylmethacrylo)dimethylsiloxy)-penta((trimethoxysilyl)ethylldimethylsiloxy)pentacyclo[9.5.1.13,9.15,15.17,13]octasiloxane)
OSS-4MA-4TMOS	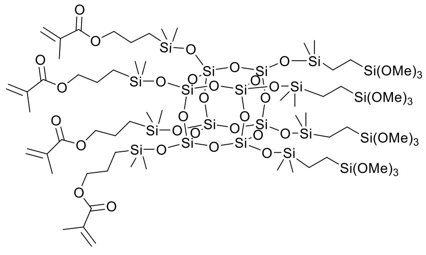	4:4	1,3,5,7,9,11,13,15-tetra((propylmethacrylo)dimethylsiloxy)-tetra((trimethoxysilyl)ethyldimethylsiloxy)pentacyclo[9.5.1.13,9.15,15.17,13]octasiloxane)

 https://doi.org/10.3390/ma18122903

**OSS-6MA-2TMOS**	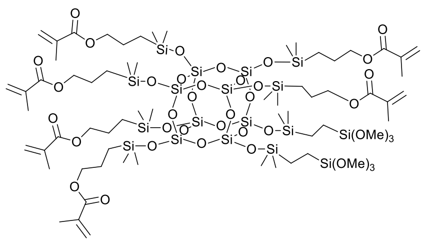	6:2	1,3,5,7,9,11,13,15-hexa((propylmethacrylo)dimethylsiloxy)-di((trimethoxysilyl)ethylldimethylsiloxy)pentacyclo[9.5.1.13,9.15,15.17,13]octasiloxane)

*MA - propylmethacryl group, TMOS - trimethoxysilyl group

Table S2. Structures of cyclosiloxane products obtained from the hydrosilylation reaction for polymer modification.

Modifier code	Chemical formula	TMOS:MA molar ratio	Name
**CS-MA-3TMOS**	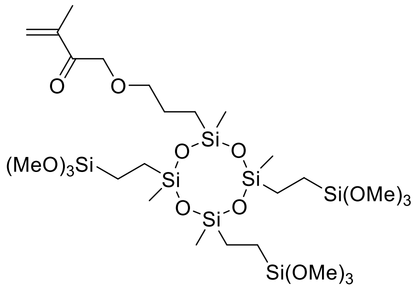	1:3	2,4,6-Tri(trimethoxysilyl)-2,4,6,8-tetramethyl-8-(propylmethacrylo)cyclotetrasiloxane)
**CS-2MA-2TMOS**	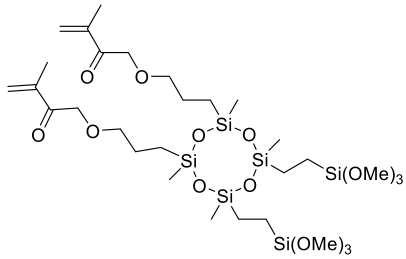	2:2	2,6-Di(trimethoxysilyl)-2,4,6,8-tetramethyl-4,8-(propylmethacrylo)cyclotetrasiloxane)

*MA - propylmethacryl group, TMOS - trimethoxysilyl group

2. NMR analytical data

2.1. CS-MA-3TMOS

2,4,6-Tri(trimethoxysilyl)-2,4,6,8-tetramethyl-8 (propylmethacrylo)cyclotetrasiloxane).

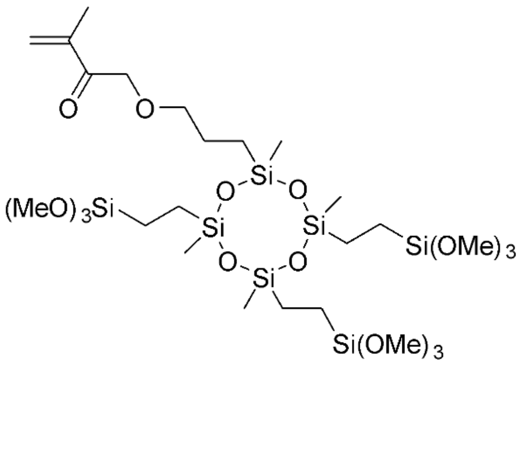

^{1}H NMR (600 MHz, $CDCl_3$) δ (ppm): 6.07 (s, 1H, C=C$\underline{H_2}$), 5.51 (s, 1H, C=C$\underline{H_2}$), 4.07 (s, 2H, O-C$\underline{H_2}$), 3.54 (s, 27H, OMe), 1.91(s, 3H, -C(=CH_2)-C$\underline{H_3}$), 1.69 (s, 2H, O-CH_2-C$\underline{H_2}$), 1.10-1.07 (m, 2H, O-CH_2-CH_2-C$\underline{H_2}$), 0.56 (s, 12H, Si-$\underline{CH_2\text{-}CH_2}$-Si), 0.17- 0.07 (m, SiMe).
^{13}C NMR (151 MHz, $CDCl_3$) δ (ppm): 167.53 (C=O), 136.63 (C=C), 125.21 (C=C), 66.93(C-O), 50.66 (Si-$(OMe)_3$), 22.47, 18.40, 18.21, 13.20, 8.04, 7.85, 7.40, 4.83, 0.48, 0.30, 0.24, -0.68, -1.51, -1.75 ($SiMe_2$).
^{29}Si NMR (119 MHz, $CDCl_3$) δ (ppm): -20,05 (SiMe), -41.72 ($Si(OMe)_3$.

2.2. CS-2MA-2TMOS

2,6-Di(trimethoxysilyl)-2,4,6,8-tetramethyl-4,8-(propylmethacrylo)cyclotetrasiloxane).

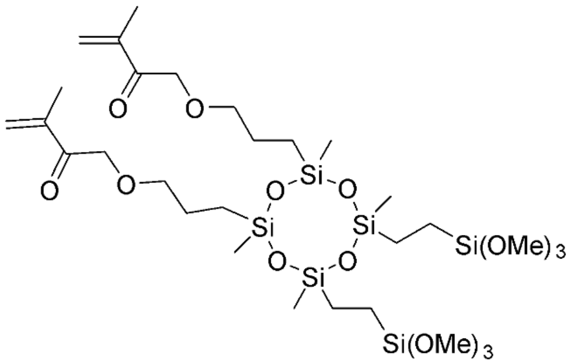

^{1}H NMR (600 MHz, $CDCl_3$) δ (ppm): 6.07 (s, 2H, C=C$\underline{H_2}$), 5.51 (s, 2H, C=C$\underline{H_2}$), 4.07 (s, 4H, O-C$\underline{H_2}$), 3.53 (s, 18H, OMe), 1.91(s, 6H, -C(=CH_2)-C$\underline{H_3}$), 1.68 (s, 4H, O-CH_2-C$\underline{H_2}$), 1.10-1.07 (m, 4H, O-CH_2-CH_2-C$\underline{H_2}$), 0.56 (s, 8H, Si-$\underline{CH_2\text{-}CH_2}$-Si), 0.17- 0.07 (m, SiMe).
^{13}C NMR (151 MHz, $CDCl_3$) δ (ppm): 167.51 (C=O), 136.59 (C=C), 128.29 (C=C), 66.89 (C-O), 50.65 66 (Si-$(OMe)_3$), 22.44, 21.51, 19.08, 18.38, 13.17, 8.01, 4.78, 0.45, -0.69,
-1.53, -3.54 ($SiMe_2$).
^{29}Si NMR (119 MHz, $CDCl_3$) δ (ppm): -20,07 (SiMe), -41.73 ($Si(OMe)_3$.

3. General overview of polymer sample preparation

3.1. Injection molding parameters

Table S3. Injection molding parameters.

Temperature (°C)	Nozzle	Zone 3	Zone 2	Zone 1	Feed
	**200**	**195**	**195**	**190**	**40**
Mold temperature (◦C)			25		
Holding pressure	t (s)		0		11
	p (bar)		700		1100
Clamping force (kN)	Holding pressure time (s)		Cooling time (s)		Screw diameter (mm)
800	11		50		25

3.2. Technological setup and product samples

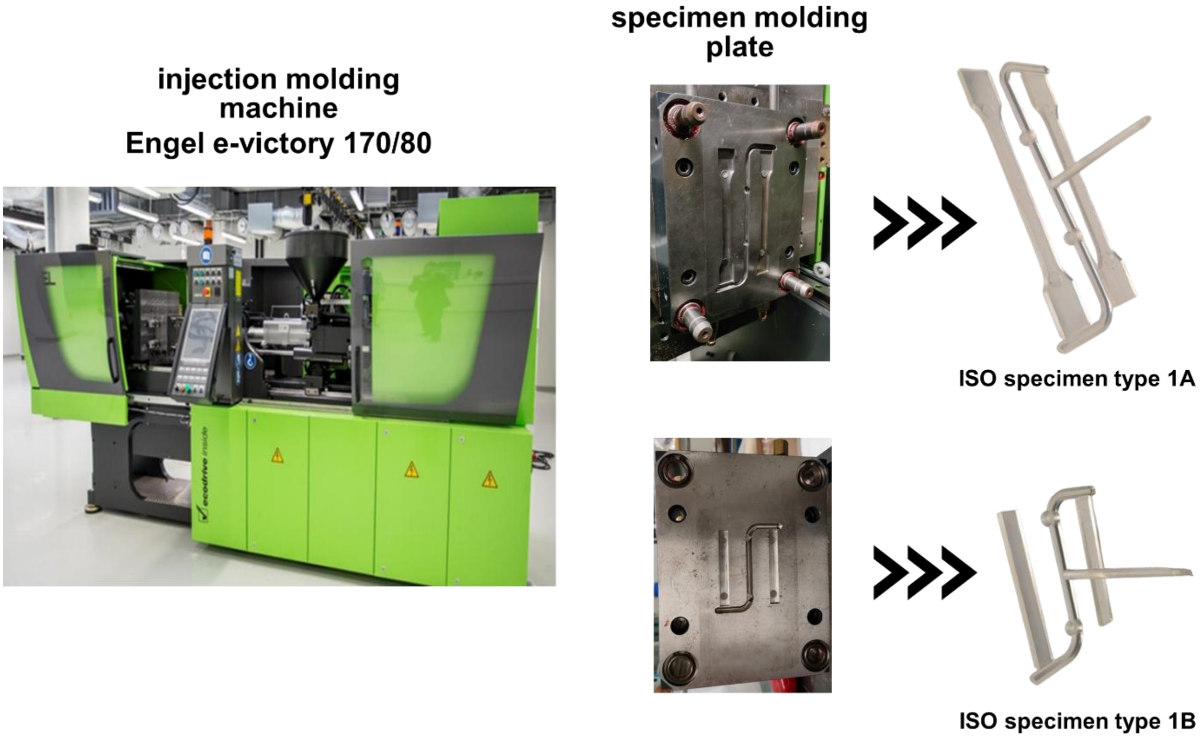

Figure S1. Injection molding set up for polylactide specimen preparation.

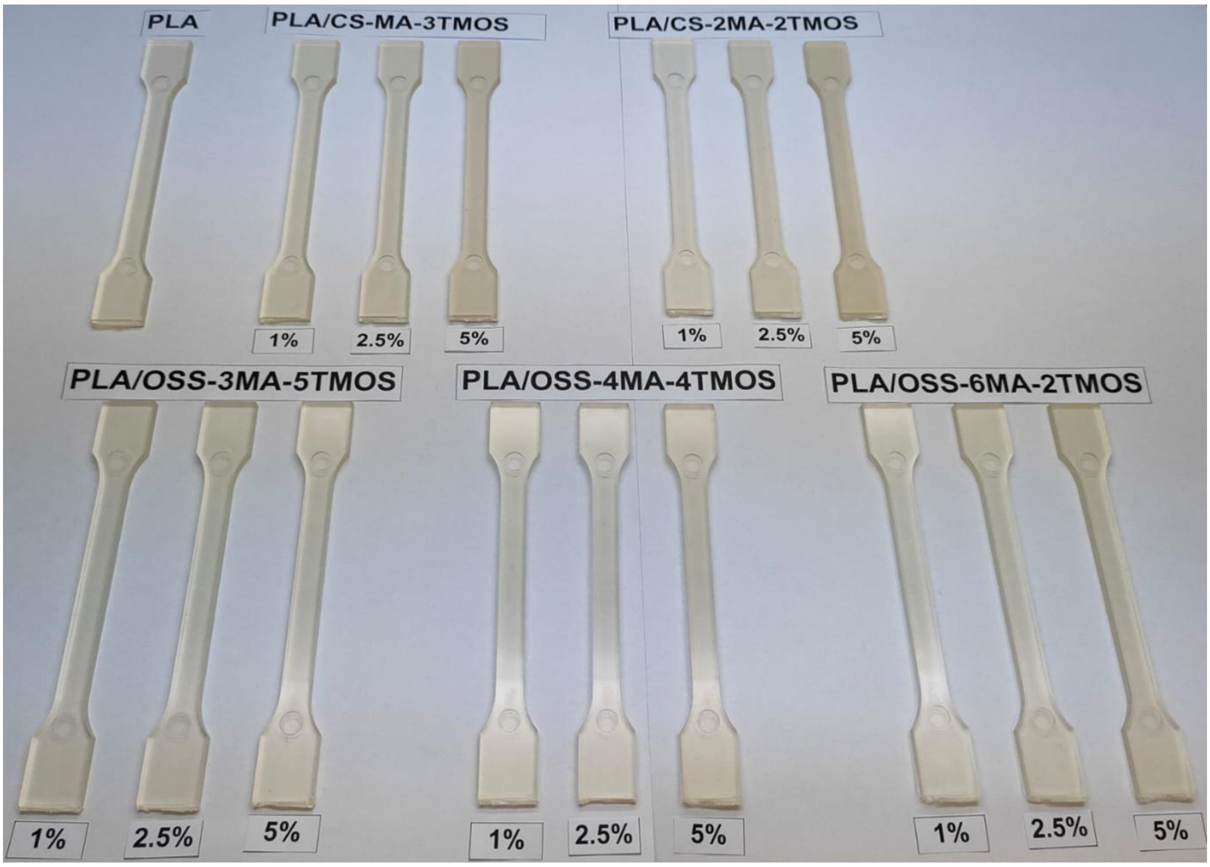

Figure S2. Influence of the modifier on appearance prepared samples - representative images of injection-molded specimens for tensile test (ISO type 1A).

4. Analytical data

4.1. SEM/EDS

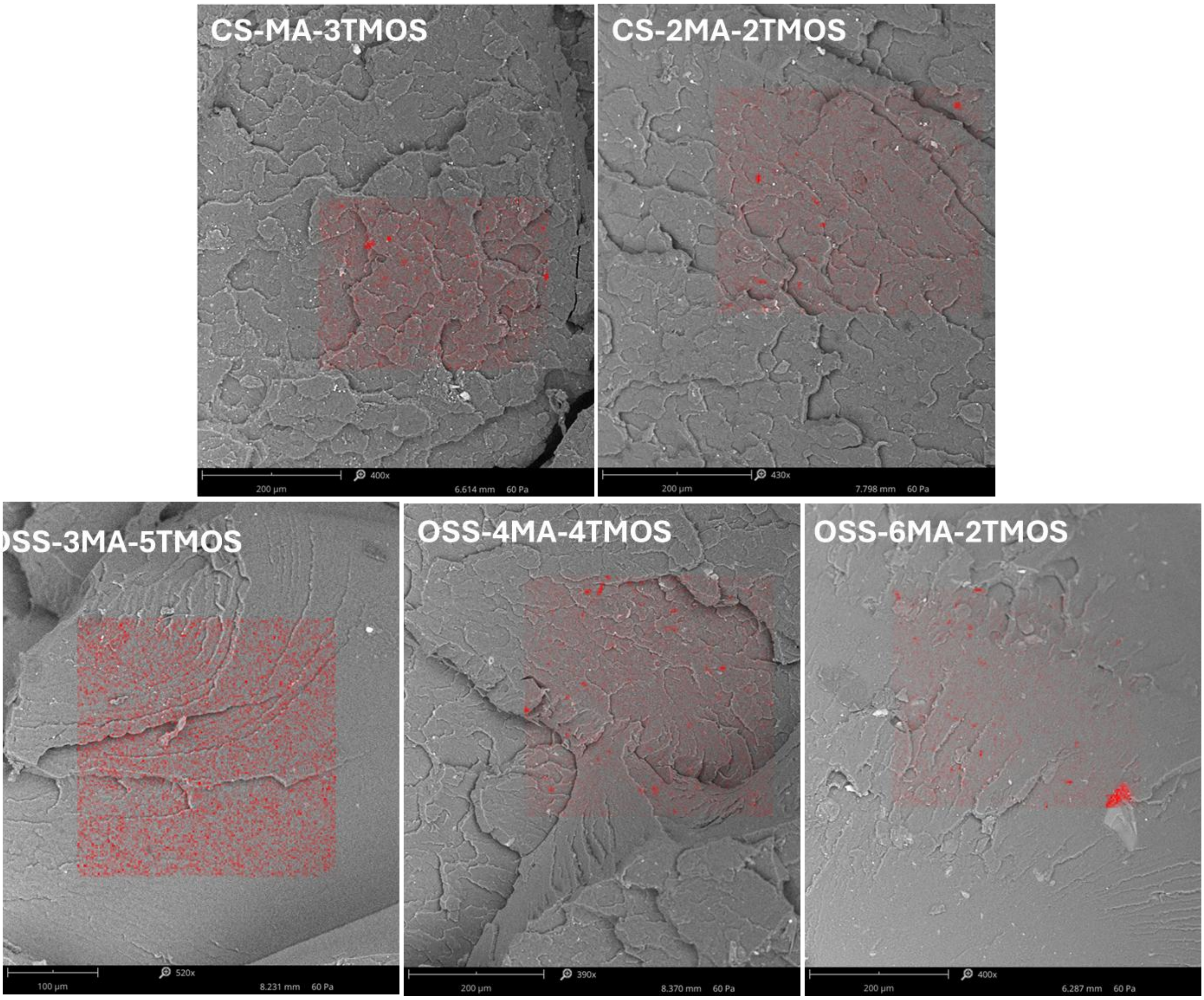

Figure S3. Organosilicone additives distribution in PLA matrix - SEM-EDS elemental mapping of Si on the breakthrough surface after impact detected for modified PLA with 5 wt% additive.

4.2. DSC

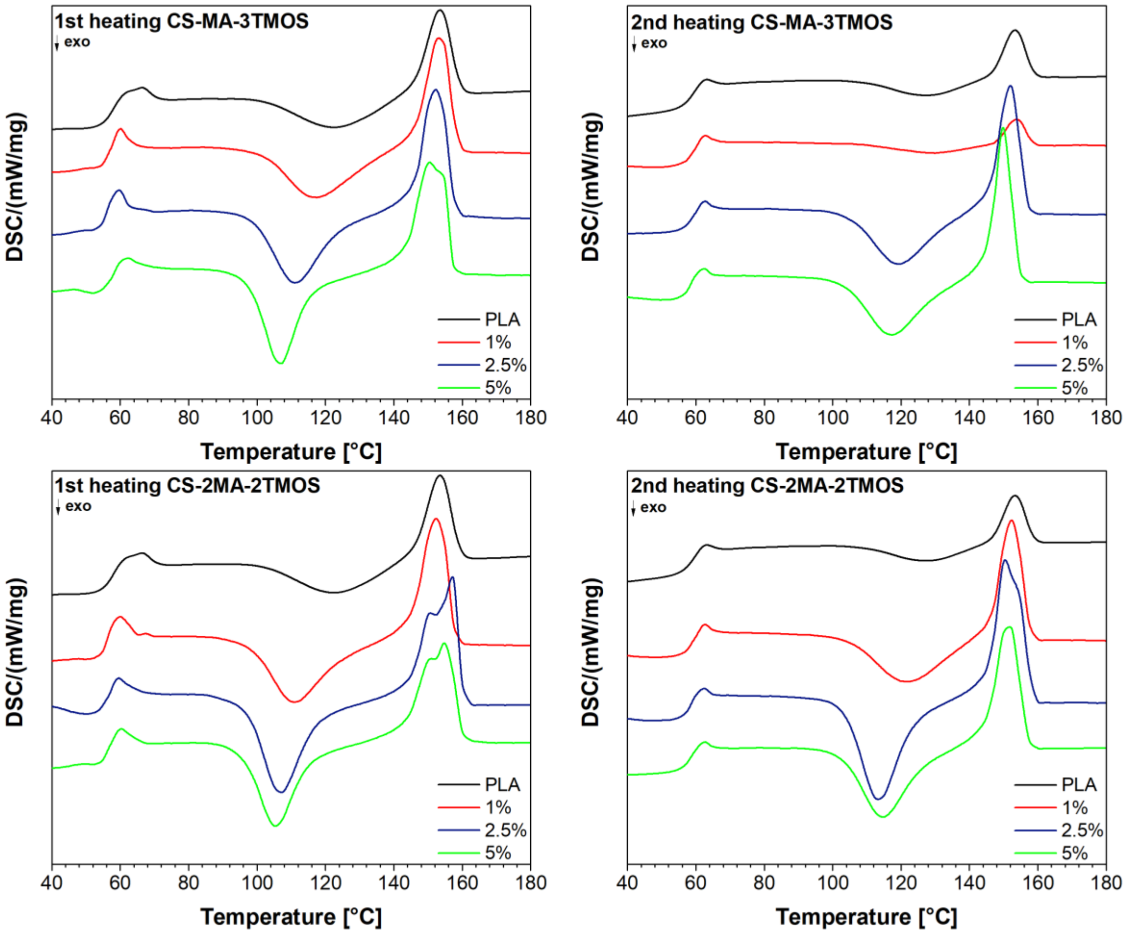

Figure S4. DSC thermograms of PLA modfyied with cyclosiloxane additives.

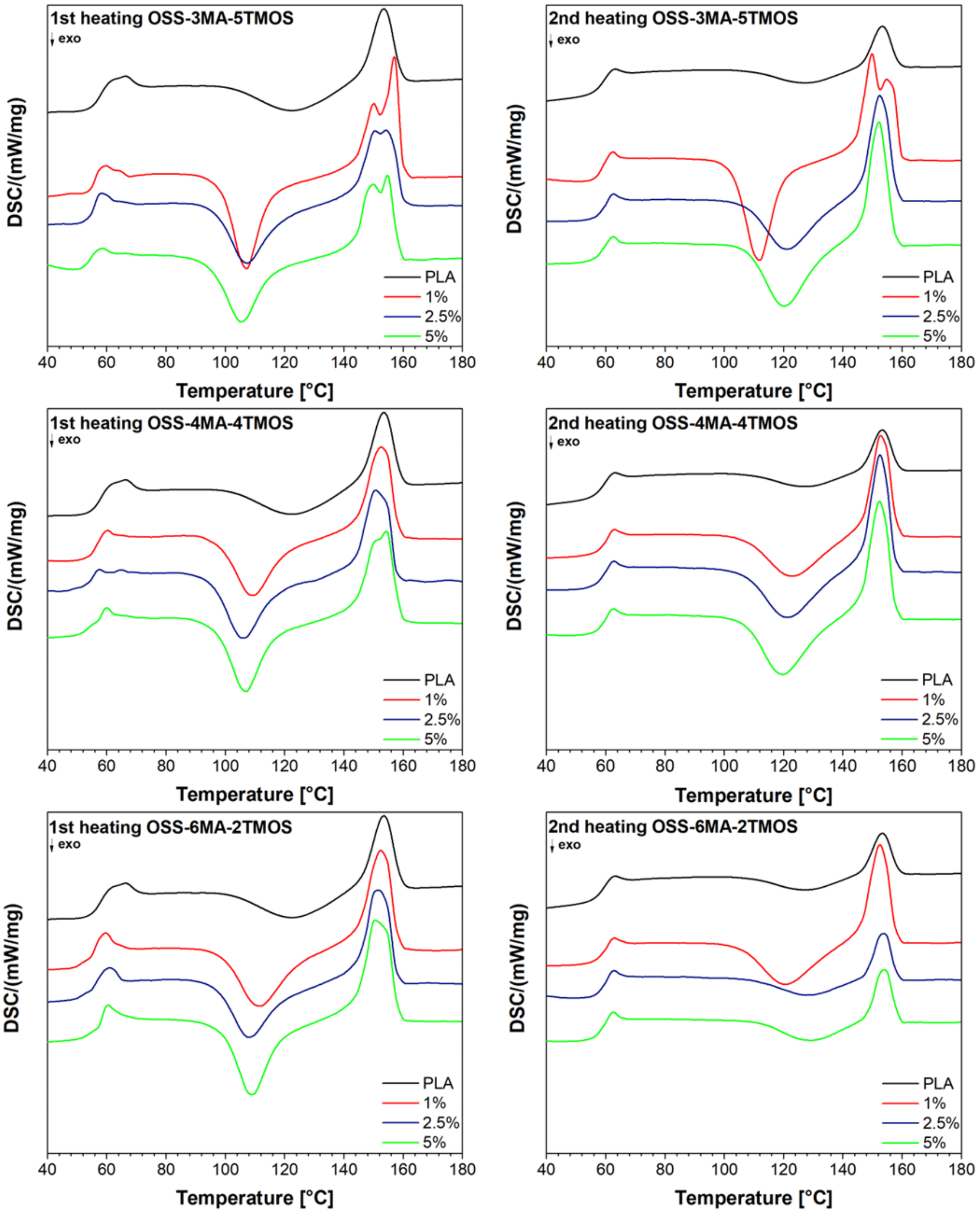

Figure S5. DSC thermograms of PLA modfyied with spherosilicate additives.

4.3. Rheology

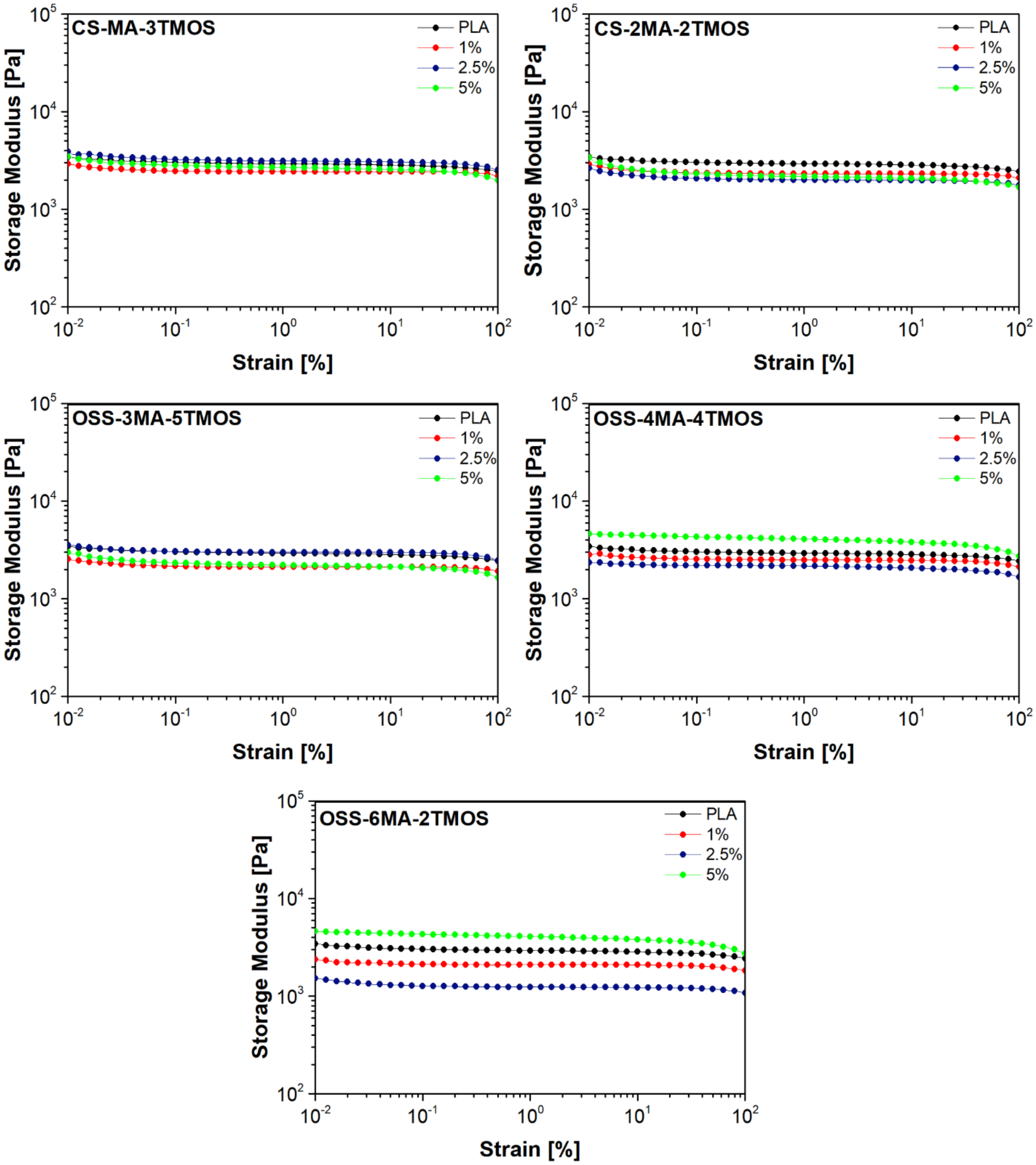

Figure S6. Strain dependence of storage modulus for PLA and modified samples at 190°C.

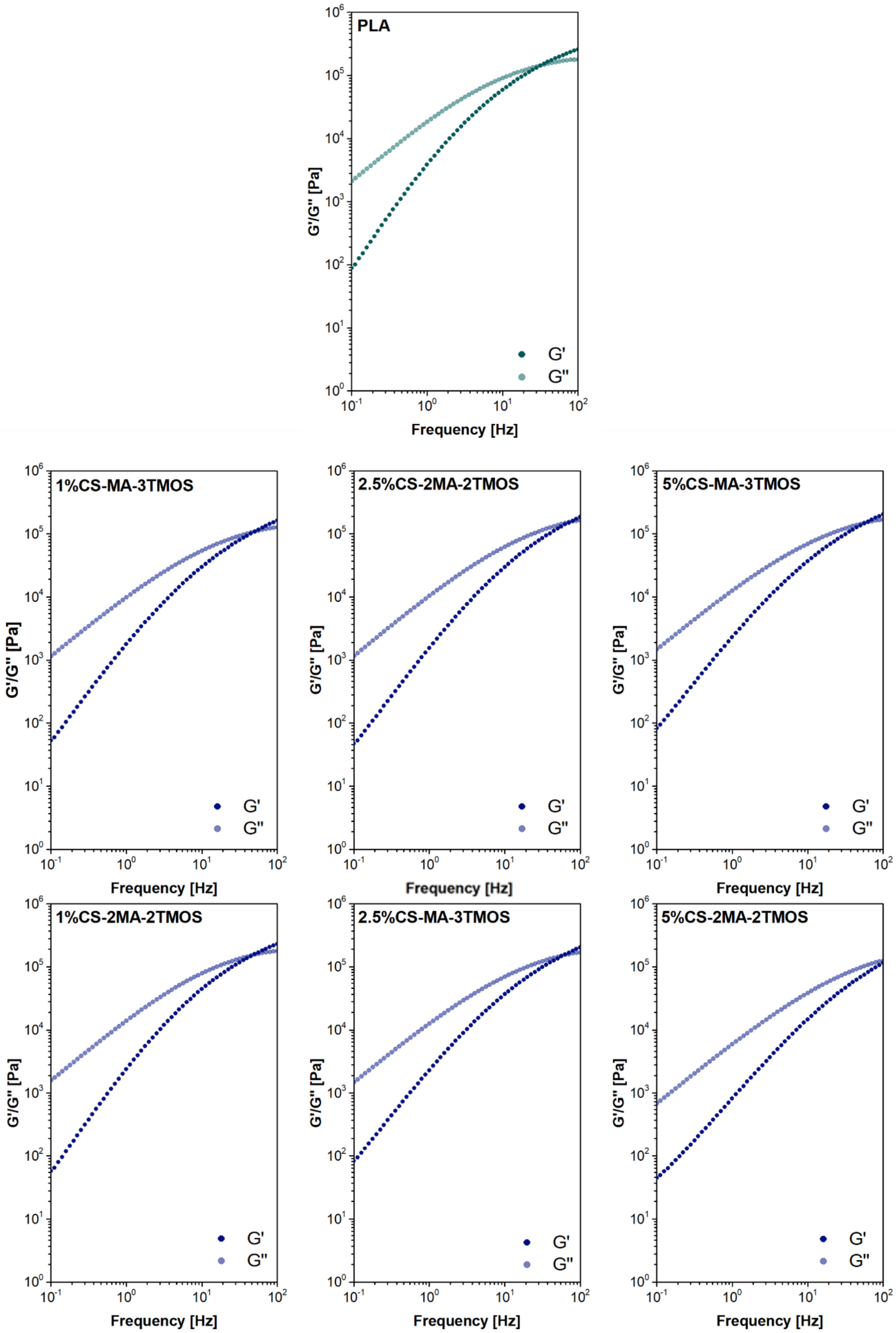

Figure S7. Storage modulus (G′) vs. loss modulus (G′′) frequency dependence at 190°C of PLA and samples modified with cyclosiloxane additives.

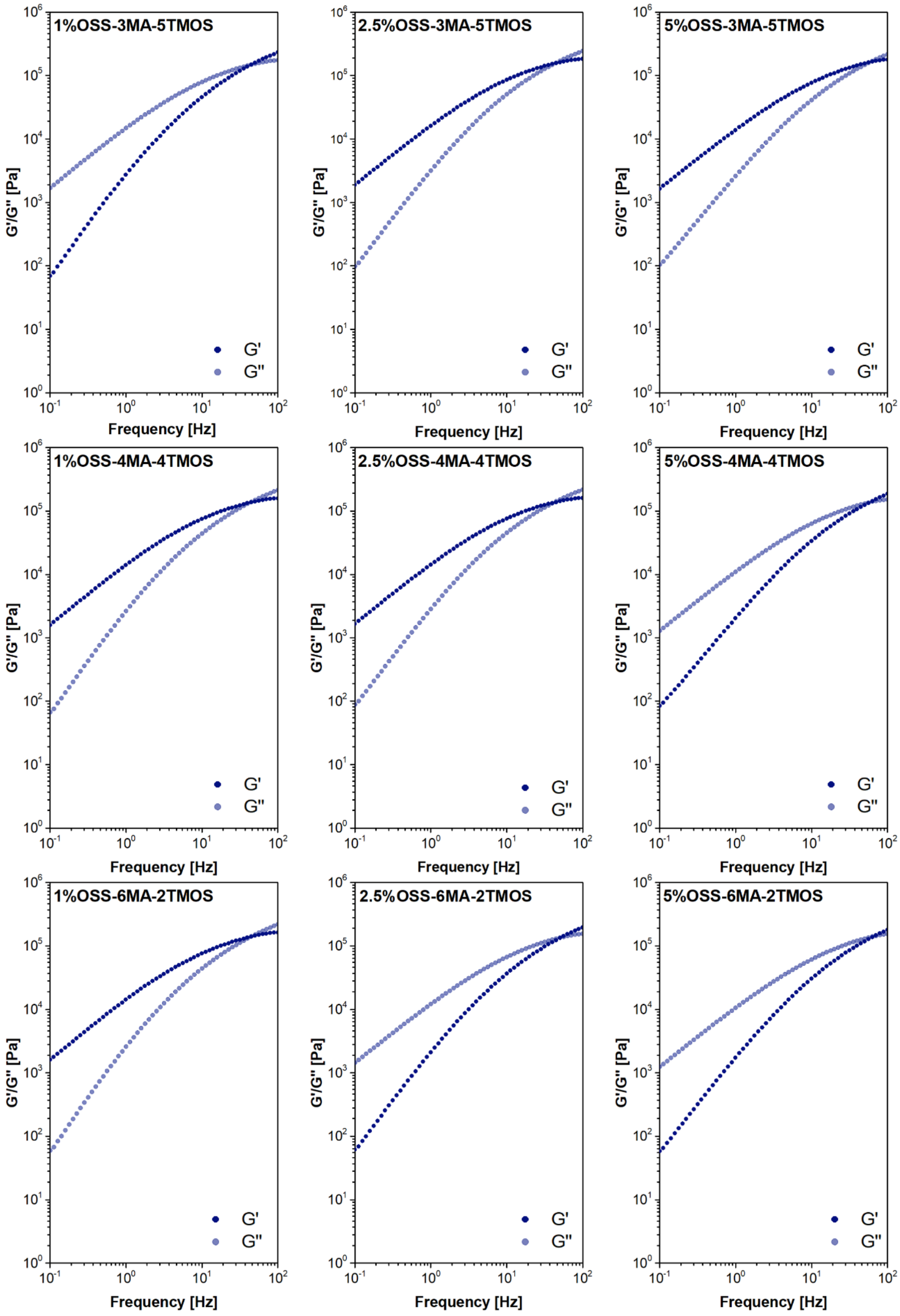

Figure S8. Storage modulus (G′) vs. loss modulus (G′′) frequency dependence at 190°C of PLA and samples modified with octaspherosilicate additives.

4.4. WCA

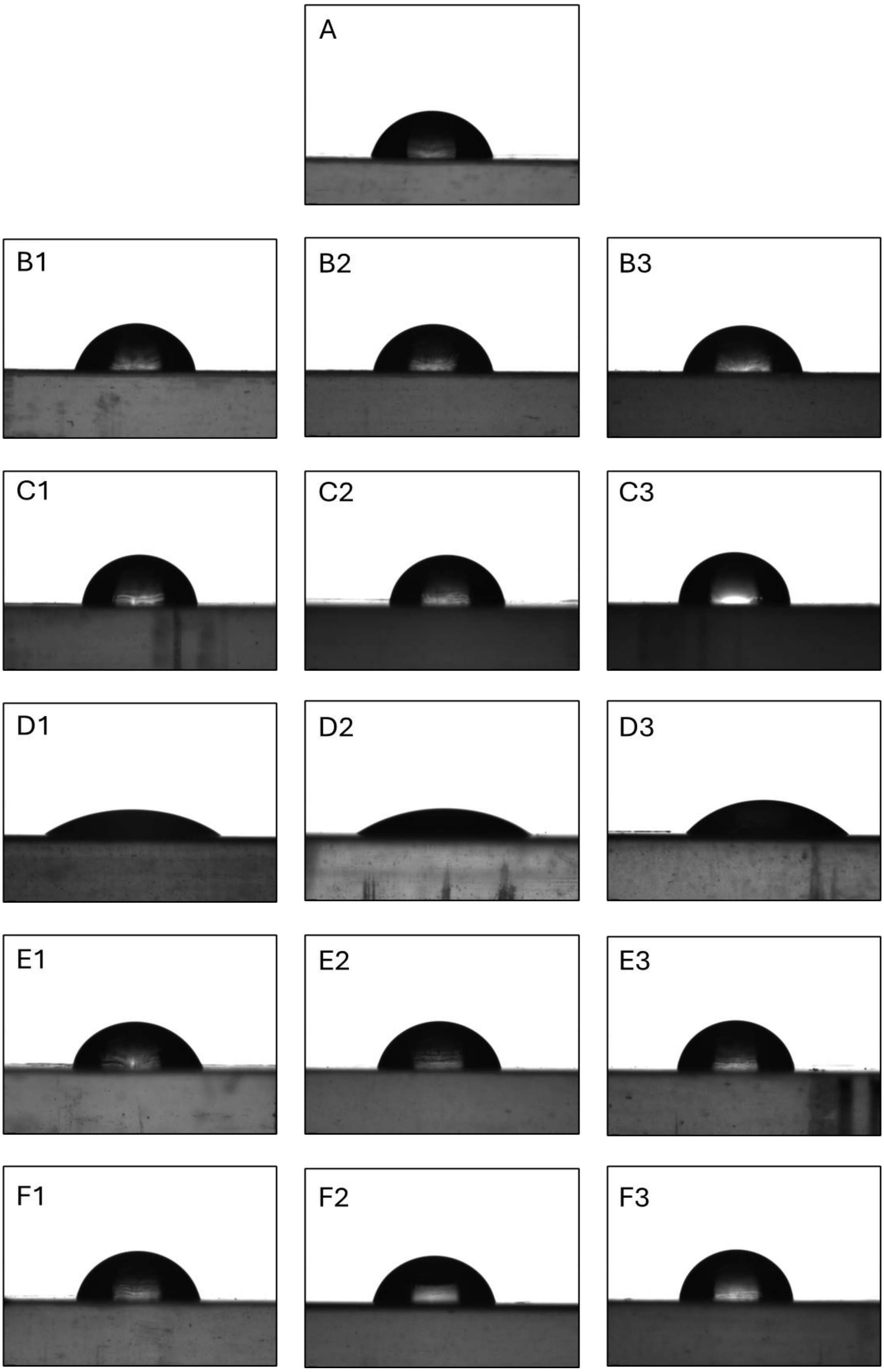

Figure S9. The pictures of a water droplet applied on the surface. A - PLA, B - PLA/CS-MA-3TMOS, C - PLA/CS-2MA-2TMOS, D - PLA/OSS-3MA-5TMOS, E - PLA/OSS-4MA-4TMOS, FE - PLA/OSS-6MA-2TMOS.
